# Socioeconomic differences and persistent segregation of Italian territories during COVID-19 pandemic

**DOI:** 10.1038/s41598-021-99548-7

**Published:** 2021-10-27

**Authors:** Giovanni Bonaccorsi, Francesco Pierri, Francesco Scotti, Andrea Flori, Francesco Manaresi, Stefano Ceri, Fabio Pammolli

**Affiliations:** 1 Impact, Department of Management, Economics and Industrial Engineering, Politecnico di Milano, Milan, Italy; 2 Department of Electronics, Information and Bioengineering, Politecnico di Milano, Milan, Italy; 3 Science, Technology and Innovation Directorate, Productivity and Business Dynamism Division, OECD, Paris, France; 4 SIT, Schaffhausen Institute of Technology, Schaffhausen, Switzerland

**Keywords:** Socioeconomic scenarios, Complex networks

## Abstract

Lockdowns implemented to address the COVID-19 pandemic have disrupted human mobility flows around the globe to an unprecedented extent and with economic consequences which are unevenly distributed across territories, firms and individuals. Here we study socioeconomic determinants of mobility disruption during both the lockdown and the recovery phases in Italy. For this purpose, we analyze a massive data set on Italian mobility from February to October 2020 and we combine it with detailed data on pre-existing local socioeconomic features of Italian administrative units. Using a set of unsupervised and supervised learning techniques, we reliably show that the least and the most affected areas persistently belong to two different clusters. Notably, the former cluster features significantly higher income per capita and lower income inequality than the latter. This distinction persists once the lockdown is lifted. The least affected areas display a swift (V-shaped) recovery in mobility patterns, while poorer, most affected areas experience a much slower (U-shaped) recovery: as of October 2020, their mobility was still significantly lower than pre-lockdown levels. These results are then detailed and confirmed with a quantile regression analysis. Our findings show that economic segregation has, thus, strengthened during the pandemic.

## Introduction

Public health strategies extensively adopted by governments at the outbreak of the COVID-19 pandemic required the implementation of non-pharmaceutical interventions (NPIs) to reduce the transmission rate of the virus^1–6^. Among NPIs, mobility restrictions proved to play a central role in decreasing social contacts^7,8^, effectively contributing to control the diffusion of several pathogens across social systems and motivating their wide adoption for the containment also of the COVID-19^9–13^.

Specifically, full national lockdowns, i.e. periods of severe mobility restrictions and social distancing for almost all citizens, were widely adopted during the first wave of the COVID-19 pandemic^14–17^, while restrictions targeted to specific geographic areas and for shorter periods constituted the preferred strategy during the second half of 2020^18–25^.

The spread of the COVID-19 was unevenly distributed among geographic areas and clustered in specific zones during the first wave of contagion^14,19,26^. However, the adoption of generalized mobility restrictions across entire national territories severely affected the whole population, causing the disruption of national mobility networks^27–29^ with relevant consequences for the socioeconomic context. The impact of the contraction of mobility flows on business activities and economic output thus sparked a vivid debate on the direct and indirect consequences of such policy measures^16,30–32^, stimulating the investigation of the socioeconomic effects of NPIs adoption^26,33,34^.

This work aims to assess the impact of NPIs by evaluating how mobility restrictions interacted with socioeconomic characteristics. In so doing, we focus on Italian territories and we rely on the assumption that a reduction in mobility flows represents a first-order proxy for the contraction of economic activities. This is plausible since a wide share of the population relies on mobility to reach workplaces, and a significant portion of productive establishments depends on direct contact with consumers to carry on their activities. For example, mobility restrictions have been shown to be strongly correlated with consumption reduction^35,36^ and loss of Gross Domestic Production (GDP)^37^ during the pandemic.

Official measurements on the economic response of individuals and firms are typically available only several months after the events take place, and often with insufficient geographic detail. Digital traces of individuals, including mobility data, are instead widely diffused, readily available and contain information on the geographic position of users^38–40^. In this work, we take advantage of a large-scale social network dataset comprising near real-time observations for over 4 million individuals, provided by Facebook through its “Data For Good” program, to measure Italian mobility patterns and assess how restrictions disrupted the corresponding mobility network. Given the severity of the COVID-19 pandemic, we follow the literature on the management of natural disasters and other disruptive events^41–44^ in which digital data have been exploited to evaluate the impact of shocks, even though these sources of information may not represent perfect survey instruments.

Extant literature on the negative impacts of NPIs on economic activities has focused mostly on the lockdown phase^26,34,45,46^, although the speed and strength of recovery after restrictions lifting have relevant consequences on the overall impact of the crisis, with potentially long-lasting effects which could modify the socioeconomic structure of a country. On a macroeconomic perspective, the study of the effects of restrictions on economic output has resulted in competing recovery scenarios from the COVID-19 pandemic^47–52^, distinguishing between a long and slow recovery (U-shaped or L-shaped), which signals the existence of long-run negative effects, and a short and fast dynamics (V-shaped), which indicates temporary disruptions.

Against this background, we investigate the existence of persistent effects from mobility restrictions in Italian territories by analyzing their recovery patterns and heterogeneous responses. In fact, some territories might limit the negative consequences of the lockdown, promptly reversing their mobility patterns once restrictions are lifted (a V-shaped recovery). Conversely, other territories might be persistently penalized by restrictions, suggesting long-lasting negative effects (a U-shaped recovery).

To investigate the existence and persistence of these heterogeneous patterns, we analyze the evolution of mobility in Italy from March to October 2020, i.e., covering both the lockdown and the recovery phases, and we construct an indicator measuring the level of mobility disruption. By ranking territories according to their mobility performance, we provide evidence on the existence of two distinct classes characterized by different behaviors. The most affected territories exhibit a slow U-shaped recovery, and at the end of our period of analysis their mobility levels are still nearly 20% lower than their pre-lockdown levels. The least affected territories have a quicker, V-shaped, recovery, with mobility reaching pre-lockdown levels around mid-July, hence only 2 months after mobility restrictions are lifted.

Then, to understand the extent to which pre-existing socioeconomic characteristics of Italian territories are informative to understand such mobility responses, we investigate the presence of commonalities among members of the two classes with both supervised and unsupervised machine learning techniques. We find significant evidence of persistent economic segregation induced by the lockdown, with most affected territories being characterized by lower income per capita and higher income inequality, in line with other recent studies^46,52–54^. Such findings are confirmed with a quantile regression analysis to assess the individual effect of different socioeconomic dimensions.

Our main variable of interest is income, but we also account for other relevant socioeconomic variables, such as the distribution of revenues per employee by productive sector, the share of employed individuals capable to work from remote, or the North-South geographic divide. Thus, we provide evidence that the effects of mobility restrictions have been unequally distributed across territories, with stronger and persistent effects where income per capita is lower.

These findings are relevant for the decision process of policymakers. The existence of two separate behaviours of recovery among territories signals the urgency of a differentiated policy response, targeting the most affected territories with intervention reducing the persistence of the effects of mobility restrictions. Moreover, our results show the relevance of pre-existing economic and social conditions for the resilience of territories during disrupting events and support governments and policymakers in tailoring effective economic relief schemes. In fact, our analysis does not simply reveal how recovery behaves differently among territories, but it pinpoints which socio-economic characteristics are reliable indicators to assess heterogeneous recovery performances.

**Figure 1:**
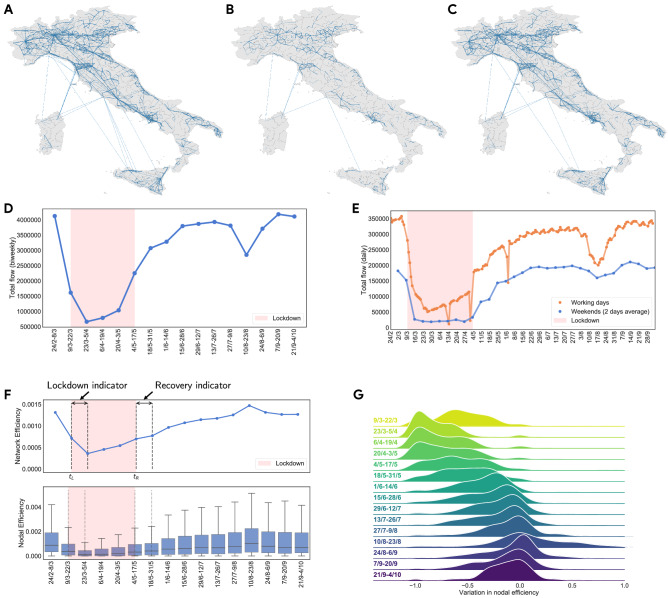
(**A**–**C**) Snapshots of Italian mobility during three distinct windows of 14-day, 24/2-8/3, 23/3-5/4, and 15/6-29/6, chosen to capture the pre-lockdown, lockdown, and recovery periods, respectively. Nodes represent LLMs and edges represent mobility flows between two locations, with thickness proportional to their weight. Figures created with Python (version 3.7.4) using *networkx* package (version 2.6.2)^55^. (**D**) Temporal evolution of the total number of individuals moving between LLMs, aggregated over windows of 14 days. (**E**) Temporal evolution of the total number of individuals moving between LLMs daily, separating working days from weekends. (**F**) Temporal evolution of the global network efficiency (top) and the distribution of nodal efficiency (bottom). We also annotate the periods in which we computed the two performance indicators, respectively Lockdown and Recovery. (**G**) Kernel density estimation of the distribution of relative variation (%) in nodal efficiency for each window w.r.t. the baseline (24/2-8/3).

## Results

### Evolution of the Italian mobility network

We collect near real-time observations of individuals moving in the Italian peninsula in a period of 32 weeks, from February 24th to October 4th, 2020, based on data of the “Disease Prevention Movement Maps” provided by Facebook through their “Data for Good” program^56^. Measurements are recorded with an 8-h frequency by aggregating individual trips of Facebook users moving between Bing map tiles^57^. Mobility flows are constructed with proprietary mechanisms to ensure privacy and anonymization, e.g., adding random noise and discarding flows with less than 10 individuals^58^. Specifically, we collect observations of traffic flows within about 5,000 distinct tiles produced by an average daily number of almost 4 million individuals, representing Italian users who enabled Facebook geolocation on their mobile phones during the period of observation.

To study the impact of variations in mobility patterns with respect to the Italian economic structure, we perform a further aggregation step by mapping tiles into 562 Italian Local Labour Markets (LLMs), i.e., territorial divisions made by the Italian Institute of Statistics (Istat) to identify uniform sub-regional areas in which the labour force lives and works, and where local firms usually employ most of their workers^59,60^ (see Fig. S5 in Supplementary Information for an illustrative example).

Using LLMs as units of analysis is particularly suitable for our study. In fact, LLMs are defined based on labor markets, i.e., areas with a homogeneous productive structure in which, therefore, we can assume internal consistency in the distribution of labor forces and commuting flows. For these reasons, we can exploit them to infer differences among comparable units. Using lower-level territorial units (such as municipalities) would have required, instead, a more complicated process of attribution of the economic productive structure since local labor markets overlap over administrative boundaries of municipalities. On the other hand, aggregating at the level of LLMs reduces our ability to capture short-range mobility flows, i.e., mobility flows *inside* LLMs. Our data, however, measure movements across fixed time windows of several hours and hence are more likely to represent long-range commuting patterns of individuals^45^, i.e., *among* LLMs, rather than shorter ones.

We then build mobility networks using a weighted undirected graph formulation, where LLMs stand for nodes, while edges are weighted by the number of individuals traveling between two nodes in a given period of time. Given the large variability of daily measurements, for instance during the weekends, we study human mobility by aggregating observations using non-overlapping windows of 14 days over the interval from February 24th to October 4th. Figure 1 provides an outlook on Italian mobility as measured by our data. Top panels (A–B–C) highlight three specific snapshots *before*, (24/2-8/3), *during* (23/3-5/4) and *after* (15/6-29/6) the national lockdown of March 8th. Middle panels show the evolution of the total number of individuals flows, aggregating either over bi-weekly windows (D) or daily (E). Note how during the lockdown period there was a strong reduction in mobility flows, consistent with the hypothesis of the disruption of the Italian mobility network.

We investigate the process of disruption on the Italian mobility network due to lockdown restrictions, along with the analysis of simple topological statistics^61,62^. More specifically, we focus on the global efficiency (see Methods) along the literature on the resilience of networks against disruptive events^63–65^. We observe a pronounced variation of several network metrics (see Figs. 1D, E and S2, S3 in the Supplementary Information) following the national lockdown, which caused the disappearance of a large number of connections and induced a fragmentation of the mobility network which is well reflected by the pattern of global efficiency in Fig. 1F. Interestingly, we also note that the strongest effects of mobility restrictions are most evident a couple of weeks after the lockdown (cf. 23/3-5/4). Finally, at the end of the period of observation (21/9-4/10), these metrics are approximately at their pre-lockdown values, signaling a return to the “business as usual” mobility levels.

When focusing on mobility disruption at the level of single nodes, we adopt the same approach as above, analyzing both simple node centrality measures, namely degree and strength^61,62^, and nodal efficiency^66,67^, a more specific measure chosen to capture local mobility disruption. Nodal efficiency is defined as the average reciprocal distance of each node from the other nodes in the network, thus representing the contribution of each node to the global efficiency of the network. Hence, this measure combines the information deriving from network cohesiveness and the distance between nodes to measure how efficiently individuals travel throughout the network (see Methods for more details). The evolution over time of the distribution of node centralities mirrors the trend of the global network statistics: a sharp decreasing trend consequent to the application of lockdown and then a gradual recovery during the summer period (see Fig. 1F for nodal efficiency, and Fig. S4 in the Supplementary Information for strength and degree centrality).

The core of our analysis relies on the assumption that mobility disruption negatively affects economic activity. We proxy shocks to mobility with the nodal efficiency of each LLM compared to the pre-lockdown scenario, i.e., the network of mobility corresponding to the first 14-day window in our sample (24/2-8/3). We consider such proxy as particularly suitable in our setting, where a complex system undergoes external stress and then recovers, because it allows us to quantify the contribution of different territories to the configuration of the network. To model the shock in mobility at node level, a good candidate is also strength centrality, which simply sums the number of individuals moving from a location to all its immediate neighbors, and it is positively correlated with nodal efficiency. However, we employ nodal efficiency because it takes the overall topology of the graph into account, thus providing a system-wide evaluation of the mobility network, while strength centrality considers only direct connections.

Hence, let *i* be an Italian LLM in our sample, we define the relative variation in nodal efficiency $\Delta e_i^t$ as $\frac{e_{i,t}-e_{i,t_0}}{e_{i,t_0}}$, where $e_i$ is the nodal efficiency of the LLM calculated in a specific window of time (see Methods), *t* represents a 14-day window and $t_0$ is the pre-lockdown window taken as reference (24/2-8/3). Figure 1G provides a Kernel Density Estimation of the variation in nodal efficiency over the entire period of observation. Note how the distribution of the variation in nodal efficiency is mainly negative during the lockdown phase, while after the release of restrictions it progressively shifts into more positive values in the upper tail of the distribution, thus capturing the effective dynamics of the human mobility network in Italy. More specifically, in the 2 weeks following the release of the national lockdown (cf. 4/5-18/5), roughly one-third of LLMs recovered at least half of their initial nodal efficiency, while at the end of our period of observation (cf. 21/9-4/10) there was still a non-negligible number of LLMs displaying negative values, thus not being able to reach the pre-pandemic levels of mobility. The distribution of variations in nodal efficiency indicates the emergence of heterogeneous responses in the recovery dynamics across LLMs, which we investigate from a micro-level territorial perspective in the following subsections.

**Figure 2:**
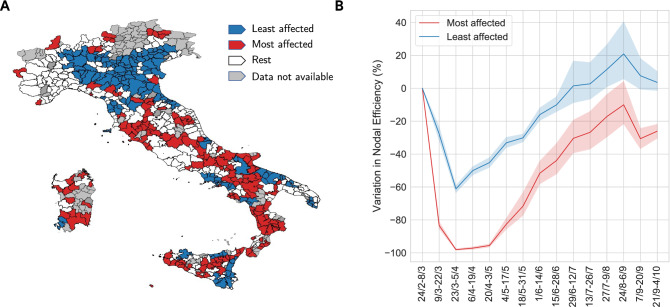
(**A**) Most (red) and least (blue) affected LLMs identified using the *lockdown* indicator and $K=30\%$. White color indicates remaining 40% of LLMs in the data, whereas grey indicates LLMs not present in our data. Figure created with Python (version 3.7.4) using *networkx* package (version 2.6.2)^55^. (**B**) Temporal evolution of variation in nodal efficiency, w.r.t. baseline (24/2-8/3), for the most (red) and the least (blue) affected LLMs, identified using *lockdown* indicator and $K=30\%$. Solid lines show mean values with 95% confidence interval (dashed area). We excluded from the plot the period corresponding to Assumption (10/8-23/8).

**Figure 3:**
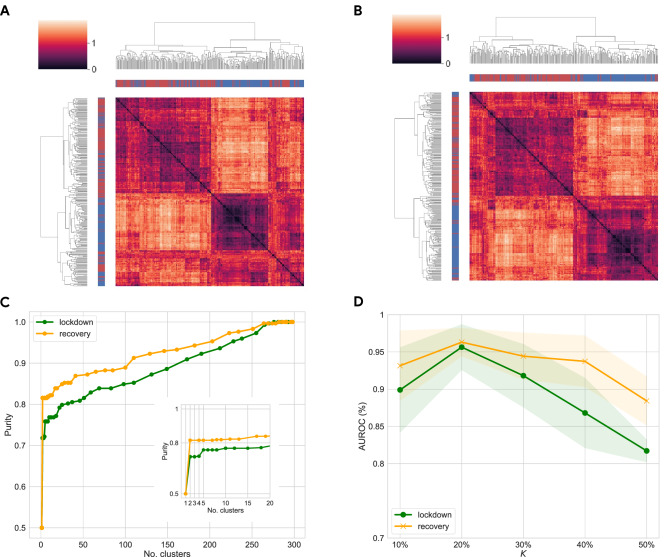
(**A**, **B**) Results of hierarchical clustering using features of the most and the least affected LLMs identified with *lockdown* (**A**) and *recovery* (**B**) indicators, with $K=30\%$. Red and blue labels of rows and columns identify respectively the most and the least affected areas. (**C**) Plot of purity score vs number of clusters for lockdown (green) and recovery (orange) dendrograms, shown respectively in panels (**A**) and (**B**). In the recovery phase, the two classes are more homogeneous and better separated, as indicated by purity scores higher compared to the lockdown period. This might signal that the effects of lockdown were more uniformly applied among LLMs, whereas recovery dynamics present more heterogeneity which allows us to better identify the most and the least affected territories. (**D**) Area under the Receiving Operator characteristic Curve (AUROC) for a Random Forest classifier evaluated on features of the most and the least affected LLMs, using lockdown (green) and recovery (orange) indicators and different values of *K* to obtain the binary label. Note the best performance for K = 20%, with AUROC values around 95% for both indicators. Similar to panel (**C**), we observe in general better results when identifying the most and the least affected LLMs during the recovery phase.

### Identifying the most and the least affected LLMs

We next examine whether the emergence of different mobility variations of Italian LLMs uncovers the presence of socioeconomic commonalities among those territories which instead share the same mobility performance in both the lockdown and recovery phases. Since socioeconomic variables do not cover the entire set of LLMs present in the mobility sample, at this stage the total number of observations in our analyses is reduced from 562 to 495 (cf. Fig. S5).

We attempt to answer the following question: do LLMs that exhibit different mobility performance, both during and after lockdown, also differ in their socioeconomic characteristics? To provide an answer, we rely on two measurements of mobility performance, one for the lockdown phase and one for the recovery phase, and we focus on those LLMS placed in the tails of the distribution of mobility performance, to identify the most and the least affected LLMs. By leveraging both unsupervised and supervised machine learning techniques, we test whether socioeconomic characteristics of LLMs are reliable predictors of their mobility performance.

We introduce two indicators that allow us to identify the most and the least affected LLMs during the lockdown and the recovery phases. We consider the nodal efficiency and denote *lockdown* as the mean percentage variation over the first two 14-day windows during the lockdown phase, and *recovery* as the mean percentage variation over the first two 14-day windows after lockdown lifting. Specifically, let $t_L$ refer to the 14-day window of lockdown (8/3-21/3), and $t_R$ to the 14-day window in which lockdown was lifted (4/5-17/5), then our two indicators of performance are computed as:

1 $$\begin{aligned} \Delta e_i^{\text {lockdown}} =&\tfrac{\Delta e_i^{t_L}+\Delta e_i^{t_{L+1}}}{2}\nonumber \\ \Delta e_i^{\text {recovery}} =&\tfrac{\Delta e_i^{t_R}+\Delta e_i^{t_{R+1}}}{2} \end{aligned}$$

Figure 1F reports the periods in which we measure the two performance indicators, while their distribution is shown in Fig. S7 of the Supplementary Information.

We then define the *most* and the *least* affected LLMs as those territories respectively in the *bottom* and in the *top* *K*% of the distribution of the aforementioned mobility performance indicators, where *K* is a threshold chosen among the bottom/top percentiles of the distribution. As shown in the Supplementary Information (cf. Figs. S8 and S9), we find high correspondence between the most and the least affected territories for different values of *K*, indicating that, regardless of which indicator is used to define the most and the least affected LLMs, our findings are robust. Besides, we find that the distribution of the variation in nodal efficiency for the two classes are well separated in almost all different periods of observation (Kruskal–Wallis (KW) $\text{ PVAL }\sim 0$ in all cases at significance level $\alpha =0.01$), with only slight overlaps in farther periods (cf. Figs. S10 and S11 in the Supplementary Information).

Panel A of Fig. 2 shows the geographical distribution of the ($K=30\%$) most and least affected LLMs identified using the *lockdown* indicator (cf. Fig. S6 in the Supplementary Information for the results obtained using the *recovery* indicator), whereas Panel B provides the temporal evolution of their mobility as expressed by the relative percentage variation in nodal efficiency. Note that the two classes experience different dynamics: the least affected LLMs exhibit a V-shaped recovery, with a steep drop followed by a fast upturn, while the most affected LLMs present a more smoothed and U-shaped pattern, with a long-lasting period of reduction in mobility and with a lag of 3 months in the recovery after the end of the lockdown. This shows the absence of a catch-up process for the most affected LLMs during recovery. Hence, not only mobility restrictions have unevenly impacted LLMs, but their effects are lasting longer in those LLMs where mobility disruption during the lockdown has been stronger. Interestingly, the dynamics of the relative percentage variation in nodal efficiency is able to capture the fact that at the end of the summer Italy experienced a strong rebound in mobility patterns, which attenuated during the early stages of Autumn showing again more penalized trajectories for the most affected territories.

**Unsupervised clustering of territories.** We employ unsupervised hierarchical clustering (see Methods) to investigate whether the *most* and *least* affected LLMs manifest distinct peculiarities in terms of socioeconomic variables.

Specifically, since the disruption of mobility is associated with the exacerbation of socioeconomic disparities^26,45,46^, we consider income per capita, inequality in the distribution of income and population density as potential drivers of variations in mobility. Furthermore, the market structure and the sectoral composition of the local economy are considered as relevant factors that might affect the impact of restrictions, given the different requirements of each sector in terms of mobility and direct contact with customers^13,33,36,68–70^. We control for overall market structure by introducing an aggregated measure representing the concentration of revenues among sectors (Revenue Concentration) in each LLM. We account for sectoral distribution of activities by including the number of employees by sector. Moreover, we control for the sectoral distribution of revenues by including the revenues per employee in each sector, i.e., a proxy of the local productivity of each sector in every LLM. Finally, we account for features affecting the response of LLMs to government interventions. Indeed, on the one hand, the Italian Government distinguished among industries having the possibility to maintain their activities open even during the lockdown (the so-called *essential* sectors) and others whose reopening was postponed. On the other hand, differences among firms in their capacity to implement work from remote influence the effect of restrictions. Hence, we account for the presence of employees in essential sectors and in sectors with higher attitudes to remote work using two aggregated variables, Essential Workers and Remote Workers, which measure the share of employees in these categories for each LLM. We use 16 aggregated NACE Rev. 2 categories to define sectors (see Table S6 for details). Overall, we consider 38 features (3 socioeconomic variables, 2 variables associated with governmental intervention and 33 variables for the sectoral composition of the economy: the number of employees and the revenues per employee for each of the 16 sectors and the overall concentration of revenues among them) measured before the lockdown and explained in detail in the Methods section (cf. Fig. S1).

Panels A and B of Fig. 3 provide results of the clustering analysis, carried out considering all aforementioned variables, to extract the two classes of territories in an unsupervised fashion (for $K=30\%$). Note how observations from the same class cluster together, as they exhibit smaller inter-distances with respect to members of the opposite class. We quantify this result through the *purity* score of each cluster. This is computed by (1) assigning each observation to the class which is most frequent in the cluster, and (2) counting the number of correctly assigned observations within each cluster and dividing by the total number of observations. We note high values of purity score in the clustering (about 90%) when extracting more than 50 clusters, with values around 70–80% when extracting as few as 2 clusters (see Panel C in Fig. 3). This suggests that the two classes exhibit a remarkable discrepancy in their socioeconomic structure. Similar results hold for $K=20\%$ (cf. Fig. S12 in the Supplementary Information).

**Binary classification of the most and the least affected territories.** We corroborate these findings by using a simple supervised machine learning framework. We aim to test whether we can accurately distinguish LLMs, belonging either to the *most* or *least* affected territories, based on their socioeconomic variables. We thus train and test several off-the-shelf classifiers, from Logistic Regression to Random Forest and K-Nearest Neighbors^71^ (see Methods). We use a stratified shuffle split cross-validation (with 10 folds and 20% of observations as test sample) to evaluate the performance of different models. We show in Panel D of Fig. 3 the Area under the Receiving Operator characteristic curve (AUROC) of a Random Forest classifier (computed as the macro average of both classes), using both *lockdown* and *recovery* indicators to extract the two classes, and different values for *K*. We note a great performance in the classification task, with AUROC values in the range 82–97%, with *lockdown* indicator performing slightly worse than *recovery*, and lower performance for both when considering larger samples (cf. $K\in [40\%, 50\%]$). Other classifiers yield similar performance (see Tables S2 and S3 in Supplementary Information), indicating that indeed mobility patterns uncover two classes which exhibit very different socioeconomic characteristics before the lockdown. We also obtain similar results when considering a period of four 14-day windows to compute the two indicators (see Fig. S13 in the Supplementary Information).

**Figure 4:**
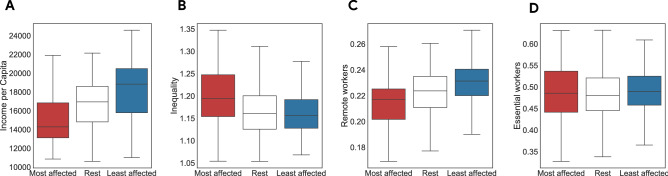
(**A**–**D**) Distributions of socioeconomic features for the most (red) and the least (blue) affected LLMs, and remaining ones (white), identified using *lockdown* variable and $K=30\%$. For variables in panels (**A**–**C**), the Kruskal–Wallis test to assess statistical differences between the distributions of the classes of LLMs is significant at a level $\alpha =0.001$. For the variable in panel (**D**), it is not significant. According to the pairwise multiple comparison tests with Conover procedure and Bonferroni correction for *p*-values, distributions are statistically different at a level $\alpha =0.01$ in all cases for variables in panels (**A**–**C**), with the following exceptions: in panel (**B**), there is no significant difference between Rest and Least; in panel (**C**) the difference between Rest and Least is significant at a level $\alpha =0.05$.

**Multiple comparison tests.** We finally check the robustness of these findings by performing pairwise multiple comparison tests, for each of these socioeconomic dimensions, among the three classes of LLMs, namely the $K=30$% most and least affected LLMs and the remaining 40% ones (see Tables S4 and S5 in the Supplementary Information). Specifically, we perform a Kruskal–Wallis (KW) test with Conover procedure and Bonferroni correction for *p*-values^72^ (see Methods). We observe that several variables exhibit statistically significant different distributions (KW $\text{ PVAL }\sim 0$ at significance level $\alpha =0.01$). In particular, we find that, using both *lockdown* and *recovery* indicators to extract classes, the most affected LLMs exhibit median values of Income per Capita and Density which are significantly smaller compared to the least affected ones, whereas median values of Inequality are higher for the most affected areas compared to the others (KW $\text{ PVAL }\sim 0$ at significance level $\alpha =0.01$, cf. Figs. 4A, B and S14, S15 in Supplementary Information). For what concerns variables describing the labour market and the industrial composition of different territories, we observe that sectors of the most affected LLMs exhibit lower levels of revenues per employee, whereas the number of essential workers does not seem to show statistically significant differences among the different classes (cf. Figs. 4C, D and S14, S15 in Supplementary Information). We remark that our analysis focuses on the differences between the most and the least affected classes (and not with respect to the rest of the territories). Hence, these tests show that although not all variables of interest exhibit a statistically significant difference between the two classes, in general territories belonging to the most or the least affected classes differ along several relevant socioeconomic dimensions.

**Table 1:** Results for quantile regression of the lockdown (top) and recovery (bottom) indicators with respect to economic, sectorial and geographic explanatory variables, including interaction of macroareas with sectorial variables.

	$q_{0.10}$	$q_{0.20}$	$q_{0.30}$	$q_{0.70}$	$q_{0.80}$	$q_{0.90}$	OLS
**Dependent variable: lockdown indicator**							
Income Per Capita	0.303$$^{***}$$	0.234$$^{***}$$	0.167$$^{**}$$	0.243$$^{**}$$	0.243$$^{***}$$	0.169$$^{**}$$	0.208$$^{**}$$
	(0.077)	(0.069)	(0.078)	(0.097)	(0.064)	(0.074)	(0.090)
Income Inequality	0.019	− 0.054	− 0.046	− 0.071	− 0.005	0.055	− 0.059
	(0.041)	(0.036)	(0.041)	(0.051)	(0.033)	(0.039)	(0.047)
Population Density	0.155$$^{***}$$	0.136$$^{***}$$	0.147$$^{***}$$	0.226$$^{***}$$	0.146$$^{***}$$	0.109$$^{**}$$	0.179$$^{***}$$
	(0.045)	(0.040)	(0.046)	(0.057)	(0.037)	(0.043)	(0.053)
Revenue Concentration	0.022	0.064	0.095$$^{**}$$	0.043	− 0.007	0.033	0.051
	(0.045)	(0.040)	(0.046)	(0.057)	(0.037)	(0.043)	(0.053)
Essential Workers	− 0.011	− 0.016	0.025	− 0.003	− 0.069	− 0.018	− 0.076
	(0.061)	(0.054)	(0.061)	(0.076)	(0.050)	(0.058)	(0.071)
Remote Workers	0.134$$^{**}$$	0.096	0.106	0.036	0.092$$^{*}$$	0.004	0.069
	(0.068)	(0.060)	(0.068)	(0.085)	(0.056)	(0.064)	(0.079)
North-Eastearn Italy	0.872$$^{***}$$	0.421	0.771$$^{***}$$	0.723$$^{**}$$	0.486$$^{**}$$	0.349	0.550
	(0.289)	(0.257)	(0.291)	(0.362)	(0.239)	(0.275)	(0.337)
Central Italy	− 0.047	− 0.095	− 0.139	− 0.264	− 0.351$$^{**}$$	− 0.030	− 0.227
	(0.199)	(0.177)	(0.200)	(0.249)	(0.164)	(0.189)	(0.232)
Southern Italy	0.455	0.215	− 0.099	0.220	0.386	0.921$$^{***}$$	0.154
	(0.285)	(0.253)	(0.286)	(0.356)	(0.235)	(0.271)	(0.332)
Insular Italy	0.237	0.147	0.071	− 0.191	− 0.247$$^{*}$$	− 1.025$$^{***}$$	− 0.253
	(0.166)	(0.148)	(0.167)	(0.208)	(0.137)	(0.158)	(0.194)
Constant	− 0.868$$^{***}$$	− 0.628$$^{***}$$	− 0.428$$^{***}$$	0.039	0.223$$^{*}$$	0.073	− 0.269
	(0.160)	(0.142)	(0.161)	(0.200)	(0.132)	(0.152)	(0.186)
AIC	1077.58	1072.87	1108.63	1261.38	1373.51	1453.74	1256.68
BIC	1325.65	1320.94	1356.7	1509.45	1621.58	1701.81	1136.68
**Dependent variable: recovery indicator**							
Income Per Capita	0.226$$^{***}$$	0.182$$^{***}$$	0.162$$^{**}$$	0.217$$^{***}$$	0.223$$^{***}$$	0.281$$^{***}$$	0.217$$^{**}$$
	(0.074)	(0.065)	(0.068)	(0.055)	(0.062)	(0.058)	(0.087)
Income Inequality	0.004	− 0.040	− 0.086$$^{**}$$	− 0.107$$^{***}$$	− 0.110$$^{***}$$	− 0.090$$^{***}$$	− 0.079$$^{*}$$
	(0.039)	(0.034)	(0.036)	(0.029)	(0.033)	(0.030)	(0.046)
Population Density	0.119$$^{***}$$	0.101$$^{***}$$	0.082$$^{**}$$	0.103$$^{***}$$	0.118$$^{***}$$	0.080$$^{**}$$	0.125$$^{**}$$
	(0.043)	(0.038)	(0.040)	(0.032)	(0.037)	(0.034)	(0.051)
Revenue Concentration	0.075$^{*}$	0.056	0.065	0.029	0.031	− 0.031	0.041
	(0.043)	(0.038)	(0.040)	(0.032)	(0.037)	(0.034)	(0.051)
Essential Workers	− 0.077	− 0.107$$^{**}$$	− 0.026	− 0.129$$^{***}$$	− 0.125$$^{**}$$	− 0.178$$^{***}$$	− 0.193$$^{***}$$
	(0.058)	(0.051)	(0.053)	(0.043)	(0.049)	(0.045)	(0.068)
Remote Workers	0.148$$^{**}$$	0.145$$^{**}$$	0.090	0.078	0.051	− 0.012	0.082
	(0.064)	(0.057)	(0.059)	(0.048)	(0.055)	(0.051)	(0.076)
North-Eastearn Italy	− 0.052	− 0.199	− 0.183	− 0.038	0.092	0.244	− 0.095
	(0.275)	(0.244)	(0.254)	(0.204)	(0.233)	(0.216)	(0.325)
Central Italy	− 0.711$$^{***}$$	− 0.793$$^{***}$$	− 0.709$$^{***}$$	− 0.706$$^{***}$$	− 0.732$$^{***}$$	− 0.690$$^{***}$$	− 0.639$$^{***}$$
	(0.189)	(0.168)	(0.175)	(0.140)	(0.160)	(0.149)	(0.223)
Southern Italy	− 0.737$$^{***}$$	− 0.845$$^{***}$$	− 0.484$$^{*}$$	− 0.376$$^{*}$$	− 0.497$$^{**}$$	− 0.634$$^{***}$$	− 0.436
	(0.271)	(0.240)	(0.250)	(0.201)	(0.229)	(0.212)	(0.319)
Insular Italy	− 0.385$$^{**}$$	− 0.381$$^{***}$$	− 0.394$$^{***}$$	− 0.869$$^{***}$$	− 1.282$$^{***}$$	− 1.938$$^{***}$$	− 1.152$$^{***}$$
	(0.158)	(0.140)	(0.146)	(0.117)	(0.134)	(0.124)	(0.187)
Constant	− 0.308$$^{**}$$	− 0.072	− 0.040	0.114	0.116	− 0.021	− 0.279
	(0.152)	(0.135)	(0.140)	(0.113)	(0.129)	(0.119)	(0.179)
AIC	1039.41	965.67	927.63	1067.09	1219.97	1348.98	1218.72
BIC	1287.47	1213.74	1175.7	1315.15	1468.04	1597.05	1098.72

Regression obtained with the Iterative Weighted Least Squares method on standardized variables. Last column shows OLS regression as a reference. Number of observations: 495. Coefficients shown for a subset of variables, full regression table reported in Tables S8 and S9 in the SI. Results with heteroskedastic-robust standard errors, obtained via wild bootstrap (1000 replications) reported in Tables S10 and S11 in the SI. $$^{*}p<0.1$$; $$^{**}p<0.05$$; $$^{***}p<0.01$$.

### The relationship between socioeconomic characteristics of territories and mobility dynamics

We finally explore the determinants of changes in human mobility of LLMs altogether, both during the lockdown and after the relaxation of restrictive measures, by using all 495 LLMs for which we have data for mobility and socioeconomic variables.

The classification analysis tells us that socioeconomic variables can predict which LLMs belong to the most or the least affected classes (i.e., a binary outcome), but it does not indicate how these factors affect variation in nodal efficiency. In this section, we propose a framework to evaluate the magnitude and, more importantly, the direction of the interplay between local mobility performances and the socioeconomic characteristics of LLMs.

The unexplored hypothesis we want to test here is the emergence *within* the two classes of peculiar relationships between the variation of mobility and the corresponding socioeconomic characteristics. For instance, among LLMs in one class the response of mobility performance to a certain variable might be negative, while among LLMs in the other class the relation might be reversed or with a reduced magnitude. Alternatively, it may happen that the correlation remains the same within the two classes, thus signaling a socioeconomic trait which strongly characterizes the mobility response of all LLMs, regardless of classes. Furthermore, by analyzing both lockdown and recovery performance of LLMs, we are interested in understanding the extent to which the impact of socioeconomic characteristics on mobility performance is stable over time, thus affecting LLMs both before and after the implementation of mobility restrictions.

To do so, we perform a quantile regression^73^ in which the dependent variable corresponds, in turn, to the *lockdown* and *recovery* indicators defined in the previous section. The quantile regression method allows us to estimate specific coefficients for the explanatory variables in each quantile of the distribution of variation in mobility, investigating the existence of different effects involving the tails of the distribution. In contrast, a traditional Ordinary Least Squares (OLS) regression, assumes a uniform relationship between the distributions of the predictors and the one of the dependent variable, providing only a single coefficient.

Our purpose is to analyze the role of socioeconomic variables on the resilience of territories. We focus, in particular, on the relationship between income per capita and mobility variation. To account for the role of other socioeconomic dimensions, we include the economic structure of territories in the econometric model and we employ a proxy of sectoral productivity (revenues per employee of each sector) to control for the influence of local productivity on the mobility performance of territories. Finally, territorial controls at the level of macroarea account for remaining unobserved local effects.

As we obtain a clear separation between the most and the least affected classes in correspondence of a threshold *K* equal to 10%, 20% and 30%, we focus on the corresponding quantiles of the distribution of the two indicators as illustrative cases. Hence, for each quantile $\tau$ of the dependent variables $\Delta e^k$, we estimate the following equation:

2 $$\begin{aligned} \Delta e_{\tau }^k =&\alpha + \pmb {\beta } \cdot \pmb {\mathrm{X}} + \pmb {\gamma } \cdot \pmb {\mathrm{Z}} \nonumber \\ k\in&\{{\text {lockdown}}, {\text {recovery}}\} \nonumber \\ \tau \in&\left[ 0.1,0.2,0.3,0.7,0.8,0.9 \right] \end{aligned}$$

where $\pmb {\mathrm{X}}=\left[ \pmb {x}, \pmb {l}, \pmb {r}\right]$ is a matrix containing the socioeconomic variables explained in the classification section. Inside $\pmb {\mathrm{X}}$, we distinguish $\pmb {x}$, a matrix of socioeconomic indicators measured at the LLM level (Income per Capita, Income Inequality, Population Density, Revenue Concentration, Essential and Remote Workers), and $\pmb {l}$ and $\pmb {r}$, two matrices with, respectively, the number of employees and revenues per employee in each sector for every LLM. Furthermore, to the first set of variables we add a matrix of territorial controls: $\pmb {\mathrm{Z}}=\left[ \pmb {z}, \pmb {z}\cdot \pmb {r}'\right]$ with $\pmb {r}' \subset \pmb {r}$. Inside $\pmb {\mathrm{Z}}$, to measure the interplay between territorial and sector variables we employ territorial controls at the level of Italian macroareas (i.e., a subdivision of the country among North-West, North-East, Center, South and Insular Italy), $\pmb {z}$, and we introduce a matrix of interaction terms, $\pmb {z}\cdot \pmb {r}'$, between territorial controls and the revenue per employee of a specific subset of sectors.

For the most relevant variables, Table 1 reports coefficients and standard errors estimated with Eq. 2, while Tables S8 and S9 in the Supplementary Information report full results. Moreover, in Tables S10 and S11 in the Supplementary Information we report results obtained with heteroskedastic-robust standard errors, obtained via wild bootstrap (1000 replications). In general, our results remain unaffected with few exceptional cases which we comment in the Supplementary Information. Next subsections are devoted to discuss separately the variables reported in Table 1, distinguishing the separate relations among the performance of territories and each group of variables: first, the main socioeconomic variables of interest; secondly, variables related to the productive structure of LLMs; and, finally, the relation captured by territorial controls.

**Main socioeconomic variables**. We observe that the coefficient of Income per Capita is positive and statistically significant for both the lower (q$_{0.10,lockdown}$ = 0.303, SE = 0.077, q$_{0.10,recovery}$ = 0.226, SE = 0.074) and upper part of the distribution of both indicators of mobility performance (q$_{0.90,lockdown}$ = 0.169, SE = 0.074, q$_{0.90,recovery}$ = 0.281, SE = 0.058). This shows that LLMs with lower Income per Capita are associated with worse mobility performance, while LLMs with higher income have better mobility performance.

The relation with Income per Capita remains almost constant between classes (i.e., for all quantiles), and both during the *lockdown* and *recovery* periods. Moreover, it is robust, since we accounted for a wide range of controls at the level of territories and sectors. This identifies Income per Capita as the main driver of our analysis and supports the hypothesis of persistence of a segregation effect by income both across territories and over time. We can safely conclude that, regardless of their class, poorer territories are always the most affected by restrictions and the slowest to recover.

Moreover, we find evidence that during the recovery phase, in the upper quantiles of the distribution of mobility the coefficient for Inequality is negative and statistically significant (q$_{0.70,recovery} = -0.107$, SE = 0.029, q$_{0.90,recovery} = -0.090$, SE = 0.030). This pattern suggests that, among LLMs in the upper quantiles of the mobility distribution, territories with higher Inequality experience a slower recovery. This result highlights the relevance of the quantile methodology: in the previous sections we have shown that inequality among the most affected LLMs is greater than the one of the least affected LLMs (cf. Fig. 4B). However, by considering all variables together and analyzing separately the two classes of LLMs, we find that inequality is more important for the recovery of the least affected LLMs.

Finally, regarding Population Density we observe a pattern similar to Income per Capita, signaling that both during and after lockdown, the mobility contraction is higher for less densely populated territories.

**Productive structure of LLMs**. We find that the aggregated share of remote workers available in each LLM has a positive and significant coefficient during both the lockdown (q$_{0.10,lockdown}$ = 0.134, SE = 0.068) and recovery phases (q$_{0.10,recovery}$ = 0.148 , SE = 0.064) for the most affected territories, meaning that among them the effect of restrictions was stronger and the recovery was slower where the pre-existing potential availability of remote workers was lower.

We also observe that the number of workers employed in essential sectors is not a key driver for mobility during the lockdown. This evidence is consistent with the results of our previous tests, where we showed that the Essential Workers variable was not statistically different between the most and the least affected LLMs (See Fig. 4D). Interestingly, we find also a negative and statistically significant coefficient across (almost) all quantiles of the distribution during the recovery. These results, rather than signaling the absence of an effect on mobility from workers in essential sectors, are instead explainable by the level of detail of our data. Our measurements of mobility, in fact, are not suited to capture brief short-range movements, occurring for instance within neighborhoods of the same city. Urban movements of this kind are those more related to essential sectors of the economy, which were allowed to operate in order to provide necessary services to citizens (as shown in other studies^13,35,36^). Since we are not able to capture the higher mobility induced by essential sectors, we do not see a significant relation during lockdown. The same phenomenon yields a negative coefficient during recovery because it causes LLMs where the share of Essential Workers is higher to show lower levels of mobility in comparison to other LLMs.

Regarding the last aggregated measure, Revenue Concentration, we find a positive and significant coefficient during recovery (q$_{0.10,recovery}$ = 0.075, SE = 0.043) among the most affected LLMs, signaling that for these territories a faster rebound in mobility is denoted by a more concentrated economy.

In addition to results on aggregated variables, we introduce sector-specific effects to reinforce our main results, i.e., matrices $\mathbf {l}$ and $\mathbf {p}$ which report Employees and Revenues per Employees for 16 NACE Rev. 2 aggregated sectors. The role of these variables is to control for the relation between mobility performance and mobility-intensive sectors and separate it from the one between mobility and income. We find coefficients in line with our expectations, i.e., we observe a positive coefficient from mobility-intensive sectors and a negative coefficient from sectors where remote work is more available. This confirms that controlling for these variables is indeed necessary. For full details, we refer the reader to Tables S8 and S9 in the Supporting Information.

**Territorial controls**. The last type of controls we introduce address the presence of territorial biases. These may affect the estimates either directly, signaling the existence of persistent differences among territories, or indirectly via an interaction with specific sectors. In the latter case, we are testing whether some industries have a different impact on mobility depending on the LLMs in which they are located.

With matrix $\mathbf {z}$, we introduce dummy variables related to the five Italian geographical macroareas using North-West of Italy as reference area, given its role during the current epidemics and relevance for the whole Italian economic system (North-West is both the richest Italian macroarea and the one most affected by COVID-19 contagion). Moreover, we use matrix $\mathbf {z}\cdot \mathbf {r}'$ to test interaction effects among such macroareas and four sectors which we deem relevant regarding mobility restrictions: Manufacturing, Constructions, Arts and Entertainment and the Health sector. We report in Table 1 the main macroarea effects and in the Supplementary Information the macroarea interaction effects (Tables S8 and S9).

Territorial controls capture unobserved effects affecting all LLMs belonging to the same macroarea. This allows us to test also the hypothesis of the role of contagion on the reduction of mobility, since the impact of the COVID-19 was unevenly distributed across Italy affecting North-Eastern and North-Western Italy more than the rest of the country, especially during the first wave of the epidemic^19,20^. While there is mounting evidence that the nexus of causality runs in the other direction, i.e., it is mobility restriction which reduces the spread of contagion^8–12^, we still perform a robustness check and look for a relation between contagion and mobility reduction. First, Figs. S16–S19 in the Supplementary Information show that the difference among the most affected and the least affected LLMs in the number of infections and deaths due to the COVID-19 is not statistically significant. Second, we find a better mobility performance in macroareas where contagion was more intense.

During the lockdown, in fact, we observe a positive coefficient only for territories in North-Eastern Italy, both among the most affected (q$_{0.10,lockdown}$ = 0.872, SE = 0.289) and the least affected LLMs (q$_{0.80,lockdown}$ = 0.486, SE = 0.239), even though with a lower coefficient. This means that LLMs in this macroarea, especially among the most affected class, had a better mobility performance with respect to the North-Western macroarea, even though the intensity of contagion among the two macroareas was comparable. On the contrary, the other Italian macroareas, where the intensity of contagion was lower, did not have a performance significantly different from North-Western. During the recovery phase, instead, we find a negative and significant coefficient of the Center, South and Insular macroareas showing a slower rebound with respect to the North-Western macroarea. This is consistent with the geographic representation of the most and the least affected LLMs reported in Fig. 2A and highlights the presence of territorial effects which, however, are not related to the spread of the virus.

Finally, regarding interaction effects between sectors and macroareas, i.e., $\mathbf {z}\cdot \mathbf {r}'$ in Eq. 2, we find that they are usually not significant (cf. Tables S8 and S9 in the Supplementary information). Among the few exceptions, it is worth focusing on the Health sector, due to its strategic role during the pandemic. In fact, we notice a positive coefficient during lockdown among least affected LLMs and across all macroareas (and especially in the insular area), signaling that those macroareas with higher concentration of revenues per employee from firms in the Health sector, experienced lower variation in mobility with respect to the reference macroarea. In the recovery phase, these effects are no longer significant.

## Discussion

In this paper, we investigate the interplay between human mobility and the socioeconomic composition of Italian territories, with a specific focus on persistent effects which characterized mobility during and after the national lockdown put in place by Italian authorities in March 2020 to prevent the spread of the COVID-19. To this aim, we leverage a massive dataset of near real-time observations of movements for approximately 4 million daily individuals in a period of 32 weeks, from March to October 2020, and we rely on detailed information on the composition of the Italian productive system by sector, using official census statistics on the number of employees and revenues, and on the socioeconomic characteristics of Italian administrations.

We show that pre-existing economic disparities among Italian territories can explain their reaction to lockdown restrictions and that two specific classes of territories emerge: the most affected and the least affected territories by interventions. The two classes show persistent behaviours, with income disparities driving their reaction both in the lockdown and recovery periods, and with income inequality playing a relevant role during the latter. Moreover, we observe that our estimates are robust once controlling for several relevant confounding factors, such as the composition of productive sectors of the economy, the share of remote workers and the share of workers from sectors exempted from restrictions by the government, and, finally, common features among territories belonging to the same geographic macroarea.

We reveal at a microeconomic level that a stable separation exists between the most affected and the least affected territories of Italy. These findings are of utmost importance for policy decisions aimed to balance the need to limit the spread of the contagion and the negative effects of restrictions on socioeconomic conditions. In particular, lockdown effects, combining with preexisting disparities, are more intense for the most disadvantaged territories, which yield a slower recovery, leading to further segregation of already deprived areas. To compensate economic losses deriving from the COVID-19 epidemic and avoid a longer path of recovery for the most penalized territories, our analysis supports the urgency of a differentiated policy response, targeting the most affected territories with intervention reducing the persistence of the effects of mobility restrictions.

## Methods

### Network efficiency

Network efficiency^63,64^ is a measure traditionally employed to study man-made and other communication networks. It combines the information deriving from network cohesiveness and the distance between nodes to assess how efficiently information/individuals travel over the network^63,64,66^.

Given a weighted undirected network *G*(*V*, *E*) with $n=|V|$ nodes and $m=|E|$ edges, the connections of *G* are represented by the weighted adjacency matrix *W* with elements $\{w_{ij}\}$, where $w_{ij} \ge 0$ $\forall$ *i*, *j*. In our analysis, we use the reciprocal of the number of individuals traveling between two locations to specify weights only when computing the efficiency measure of the network (and the nodes). Given that our network resembles a commuting network, we assume that two locations are closer if many individuals travel between them. The global network efficiency is thus computed as:

$$\begin{aligned} E_{glob}(G) = \frac{1}{n(n-1)}\sum _{i \ne j \in G} \frac{1}{d_{ij}} \end{aligned}$$

where $d_{ij}$ is the shortest path distance between two generic nodes *i* and *j*. When nodes *i* and *j* cannot be connected by any path, then $d_{ij} = +\infty$.

Following the methodology of Latora et al.^63^ , $E_{glob}(G)$ is normalized in order to assume the maximum value in the case of perfect efficiency, i.e., in a fully connected graph where all nodes are connected to each other via the shortest possible distance. In our framework, this corresponds to the shortest distance between two nodes in the network, namely we assume that $\min (d_{ij})=\min (w_{ij})=w_{\min }\, \forall i,j$. The normalized global efficiency can be thus computed as:

$$\begin{aligned} E_{norm}(G)=\frac{E_{glob}(G)}{E_{ideal}(G)} = E_{glob}(G) \cdot w_{min} \end{aligned}$$

where $E_{ideal}(G) = \frac{1}{n(n-1)}\sum _{i \ne j \in G} \frac{1}{w_{min}} = \frac{1}{w_{min}}$. The shortest distance between two nodes, namely $w_{\min }$, varies depending on the time window of 14 days in which the network is built.

In addition, we define the nodal efficiency as the contribution of each node *i* to $E_{glob}(G)$:

$$\begin{aligned} e_{i} = \frac{1}{n-1}\sum _{i \ne j \in G}\frac{1}{d_{ij}}, \end{aligned}$$

and, accordingly, the normalized nodal efficiency of node *i* as $e_{i}^{norm}= e_i\cdot w_{\min }$.

### ASIA database

The Italian National Registry of Active Firms (ASIA) is managed by Istat and reports information on all economic units on the national territory exercising industrial, commercial and services activities. It collects and updates annually identification information (name and address) and structure (economic activity, dependent and independent employees, legal form, date start and end activities, turnover) of firms within the register. Even though the data is collected at a granular level, for privacy reasons it has been provided to us by Istat at the province level and for each NACE Rev. 2 business sector which we aggregate in 21 macrocategories (see Table S6 in the Supporting Information for details).

### Matching procedure for economic variables

Our analyses integrate data measured at the municipal level with information regarding the productive structure of the Italian economy at the level of provinces, i.e. at a more aggregate level. In order to ensure a reliable number of observations we bridge the two datasets towards the intermediate level of analysis of Local Labour Market (LLMs) which are comparable sub-regional geographical areas defined by the Italian National Institute of Statistics (Istat). As a result, our economic data cover different dimensions of the local socioeconomic context of each Italian LLM, in total 495 areas. Our three main variables of interest are Income per Capita, Inequality in the distributions of incomes and Population Density. These variables have been collected at the municipal level and then aggregated at the level of LLMs mapping each Italian municipality to a unique LLM. We then integrate these variables with sector related ones (employees and revenues per employee) obtained from the ASIA database at the province level. Using publicly available data from Istat on the number of employee at municipal level, we were able to redistribute province level data to the LLM level of aggregation using the share of province employees for each municipality as partition coefficient and the municipality-to-LLM mapping to aggregate the municipality data.

### Description of economic variables

**Declared income**. This variable is the tax base of the personal income tax declared by tax payers in the 2018 tax return to the Revenue Agency for the 2017 financial year. The distribution at the municipal level is provided by the Ministry of Economic and Finances (MEF) and can be recovered from Istat. We calculate Income per Capita as the ratio of total declared taxable income in a municipality divided by number of declarants with taxable income.

**Income inequality**. We measure income inequality at the municipal level as the ratio between average and median values of the distribution of the declared income, without taking into account cadastral incomes. If the distribution of income is skewed, as is the case for 98% of Italian municipalities, the average municipal income will be consistently greater than the median municipal income. Hence municipalities with greater mean to median ratio will have a more unequal distribution than municipalities with lower ratio.

**Density of population**. We use population density to account for concentration individuals in a certain territory. It is calculated as the ratio of total population calculated at the first of January divided by the extension of the territory in squared kilometers.

**Concentration of revenues by sector**. We calculate this variable as the Herfindahl–Hirschman concentration index of the revenues of sectors in each LLMs. Let us define the revenue of LLM *i* in sector *j* as $r_{ij}$ and all its revenues $R_i$ then the HHI of each LLM is defined as $HHI_i=\sum _j \left[ \frac{r_{ij}}{R_i}\right] ^2$.

**Percentage of workers who are able to work from remote**. We use estimates of remote workers by sector from^74^ and adapt them to Italian firms. We obtain the LLMs share of potential remote worker as the weighted sum of each sector employees number multiplied by each sector-specific remote share estimate. The obtained estimates are also in line with those of^75^.

**Percentage of workers in sectors declared essential by the government **. Essential sectors are obtained from official Government decrees. We obtain the LLMs share of essential sector workers as the percentage of employee from essential sectors over all employees from all sectors.

**Territorial controls**. Our regression sample provides a good representation of the entire set of Italian municipalities, both in terms of population size and geographical location, including 2,387 observations corresponding to 30% of Italian municipalities. Nevertheless, to exclude spatial spillovers effect we control for territorial confounding factors using macroarea controls corresponding to NUTS1 areas, which represent the most aggregated geographical classification of national administrative units provide by Eurostat (see Table S7 in the Supporting Information for details). We use macroarea controls to make the estimation of interaction effects in our regression easier and avoid inflating the regression with too many covariates.

### Clustering, classification, multiple test analysis and quantile regression

We rely on several open-source packages to perform our analysis of the most and the least affected territories. We encode each LLM with a vector of socioeconomic covariates (see previous section) and we assign them a binary label, based on the *lockdown* and *recovery* indicators defined in the main text.

We employ *scipy*^76^ to carry out a hierarchical clustering of LLMs based on their standardized socioeconomic variables, using a cosine distance and average linkage to obtain clusters. We report similar results when using euclidean distance or other linkage approaches, e.g., complete or Ward linkage.

We use *scikit-learn*^77^ to train several binary classifiers, namely Logistic Regression, Support Vector Machine, Random Forest and K-Nearest Neighbors, in the task of classifying most vs least affected LLMs based on their socioeconomic variables. We apply standardization of features and use default hyperparameters as specified in the package, and we use stratified shuffle-split cross-validation to evaluate their performance.

We employ *scikit-posthocs*^78^ to perform multiple test comparisons and assess statistical differences in the distribution of features of the most and the least affected LLMs (and remaining ones). In particular, we apply Kruskal–Wallis test with Conover procedure and Bonferroni correction for *p*-values.

Finally, all quantile regression results have been performed with the *quantreg* R package^73^ on standardized variables with default options. Results in the Supplementary Information are obtained with heteroskedastic robust standard errors obtained via wild bootstrap with 1000 iterations.

## Supplementary Information

Supplementary Information 1.

## Data Availability

Data describing movements of individuals in Italy during the period of observation were provided under an academic license agreement with Facebook through its “Data for Good” program (available at: https://dataforgood.fb.com/tools/disease-prevention-maps/). All observations are released in an aggregated and de-identified manner to ensure privacy and anonymization of single users. Origin-destination matrices which describe traffic between tiles in non-overlapping windows of 14-day can be provided upon request to the corresponding author. With the only exception of the median income used to calculate the inequality index, all economic variables have been provided by the Italian National Institute of Statistics (I.stat). Municipal data on income, population and employee by sector are all available from the Istat website (http://dati.istat.it/). Data at the province level regarding revenues by sector have been obtained from the National Registry of Active Firms (ASIA database) and are available to researchers upon request. Finally, median income has been provided by the Italian Ministry of Economy and Finances (MEF). For further details we refer the reader to Table S1 in the Supporting Information.
