# Supplementary material for: Socioeconomic differences and persistent segregation of Italian territories during COVID-19 pandemic
Source: Sci Rep. 2021 Oct 27;11:21174. doi: 10.1038/s41598-021-99548-7 (PMC8551210; doi:10.1038/s41598-021-99548-7)
Supplement: Supplementary file 1 — Supplementary Information 1. [file 41598_2021_99548_MOESM1_ESM.pdf]

Supplementary Information for: Socioeconomic differences and persistent segregation of Italian territories during COVID-19 pandemic

Giovanni Bonaccorsi[1,*,+], Francesco Pierri[2,+], Francesco Scotti[1,+], Andrea Flori[1], Francesco Manaresi[3], Stefano Ceri[2], and Fabio Pammolli[1,4,]**

[1]Impact, Department of Management, Economics and Industrial Engineering, Politecnico di Milano
[2]Department of Electronics, Information and Bioengineering, Politecnico di Milano
[3]OECD, Science, Technology and Innovation Directorate Productivity, Innovation and Entrepreneurship Division
[4]SIT, Schaffhausen Institute of Technology, Schaffhausen
[*]giovanni.bonaccorsi@polimi.it
[**]fabio.pammolli@polimi.it
[+]these authors contributed equally to this work

ABSTRACT

Lockdowns implemented to address COVID-19 pandemic have disrupted human mobility flows around the globe to an unprecedented extent and with economic consequences which are unevenly distributed across territories, firms and individuals. Here we study socioeconomic determinants of mobility disruption during both the lockdown and the recovery phases in Italy. For this purpose, we analyze a massive data set on Italian mobility from February to October 2020 and we combine it with detailed data on pre-existing local socioeconomic features of Italian administrative units. Using a set of unsupervised and supervised learning techniques, we reliably show that the least and the most affected areas persistently belong to two different clusters. Notably, the former cluster features significantly higher income per capita and lower income inequality than the latter. This distinction persists once the lockdown is lifted. The least affected areas display a swift (V-shaped) recovery in mobility patterns, while poorer, most affected areas experience a much slower (U-shaped) recovery: as of October 2020, their mobility was still significantly lower than pre-lockdown levels. These results are then detailed and confirmed with a quantile regression analysis. Our findings show that economic segregation has, thus, strengthened during the pandemic.

Contents

1 Data **2**
2 Topological analysis of Italian mobility network **3**
3 Identifying the Most and Least affected LLMs **7**
3.1 Robustness checks for labeling criteria . . . 9
3.2 Clustering . . . 12
3.3 Classification . . . 13
3.4 Class comparison . . . 15
3.5 Multiple comparison tests . . . 16
4 Exploratory analysis of the interplay between human mobility and epidemiological variables **19**
5 Additional codes **24**
6 Quantile regression results **26**
6.1 Results with default standard errors . . . 27
6.2 Results with standard errors obtained via wild bootstrap . . . 35
References **43**

1 Data

For economic variables, we provide the appropriate references in Table 1. We also provide the distribution of values for the following variables in Figure 1: Income per Capita, Inequality, Fraction of essential workers, Density, HHI0, Fraction of remote workers.

	Calculation	Source	URL
Variation in nodal efficiency	Rate of change over time of the efficiency calculated on Facebook mobility networks	Facebook Data For Good program	https://dataforgood.fb.com/tools/disease-prevention-maps/ (available upon request)
Income per Capita	Total declared income divided by total number of declaring individuals	Istat	http://dati.istat.it/Index.aspx?DataSetCode=MEF_REDDITIIRPEF_COM
Inequality	Relative difference between mean and median income	MEF	Researchers can obtain it from the Italian Ministry of Economy and Finances (MEF)
Population Density	Number of individuals per square kilometer	ISTAT	http://dati.istat.it/Index.aspx?DataSetCode=DCIS_POPRES1, http://dati.istat.it/Index.aspx?DataSetCode=DCCV_CARGEOMOR_ST_COM

Table 1. Available sources of data with description of the transformation process adopted

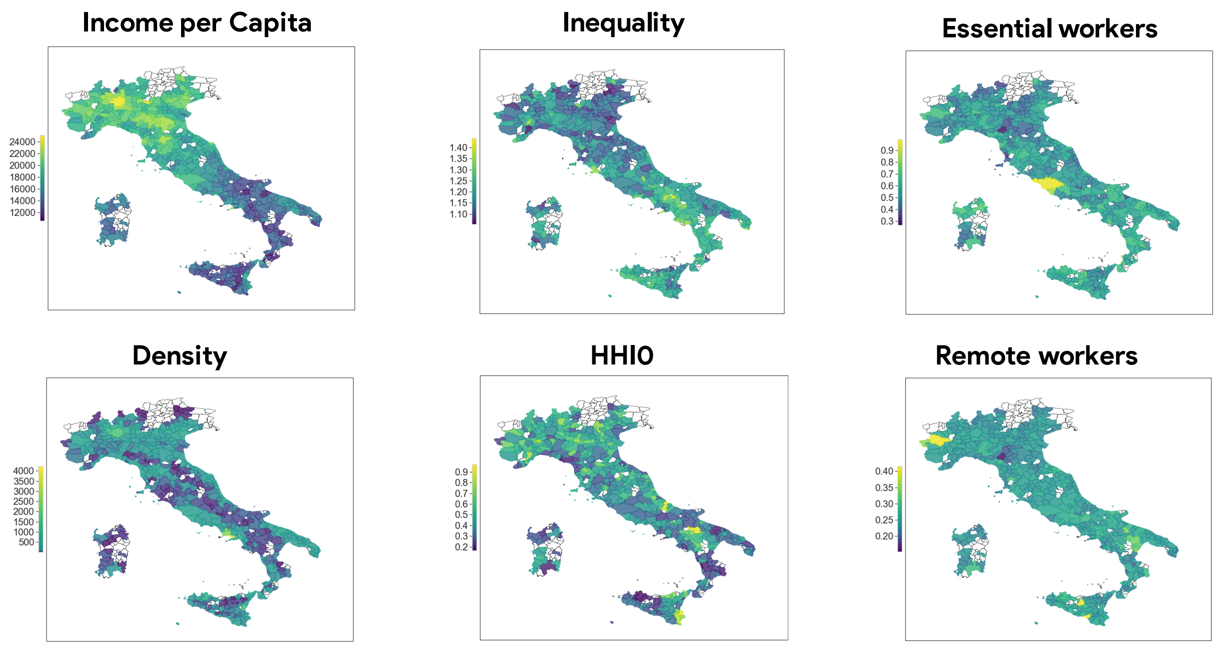

Figure 1. Distribution of several socioeconomic variables for LLMs in our dataset. Colorbar is centered on the median value.

2 Topological analysis of Italian mobility network

In this section we provide plots for the temporal evolution of several topological indicators[1,2] which allow us to investigate the dynamics of human mobility in Italy.

In Figure 2 we show the assortativity, clustering coefficient (CC), density, size of the giant connected component (in % w.r.t the total number of nodes), number of edges, number of connected components and size of the top-10 biggest connected component.

In Figure 3 we show the Jaccard distance computed between mobility graphs built on different time windows, based on the set of edges present at each period.

In Figure 4 we show the distribution of degree, strength and betweenness for each window of 14 d. Overall, we observe a similar pattern for these metrics, as the national lockdown induced a strong reduction in the distribution of all the three centrality indicators, which assume the lowest values in the bi-weeks 22/3-5/4, 6/4-19/4 and 20/4-3/5. Moreover, we highlight that at the end of the analysed timeframe (21/9-4/10), these metrics account for values that are similar to those assumed before the lockdown.

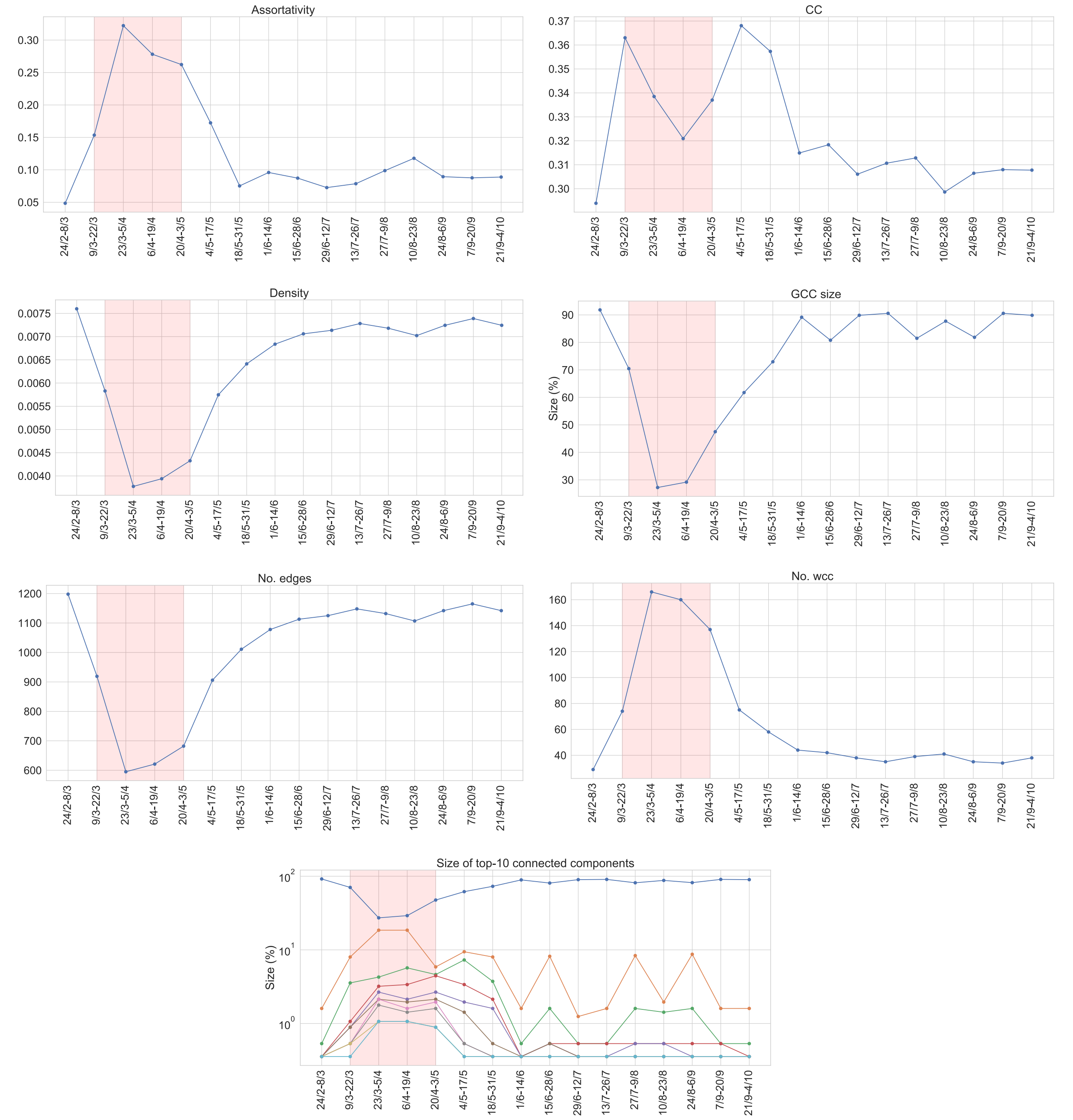

Figure 2. Temporal evolution of topological properties computed on mobility networks aggregated over windows of 14 days. Red indicates lockdown period.

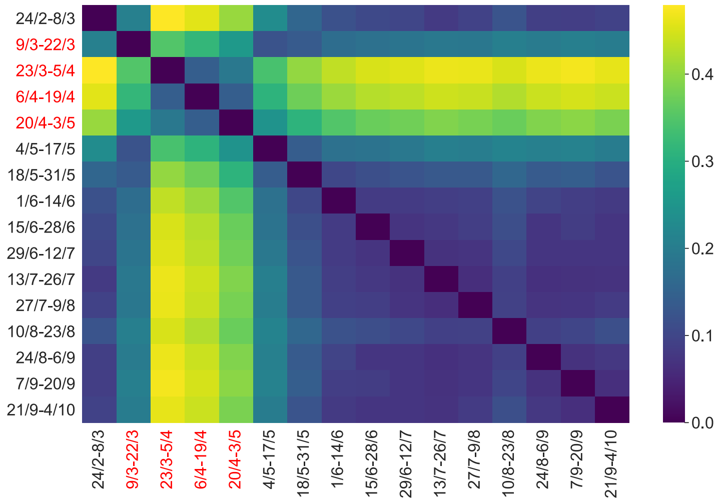

Figure 3. Heatmap that shows the Jaccard pairwise distances between networks based on the set of edges present in each window of 14 days. Brighter colors indicate higher dissimilarities.

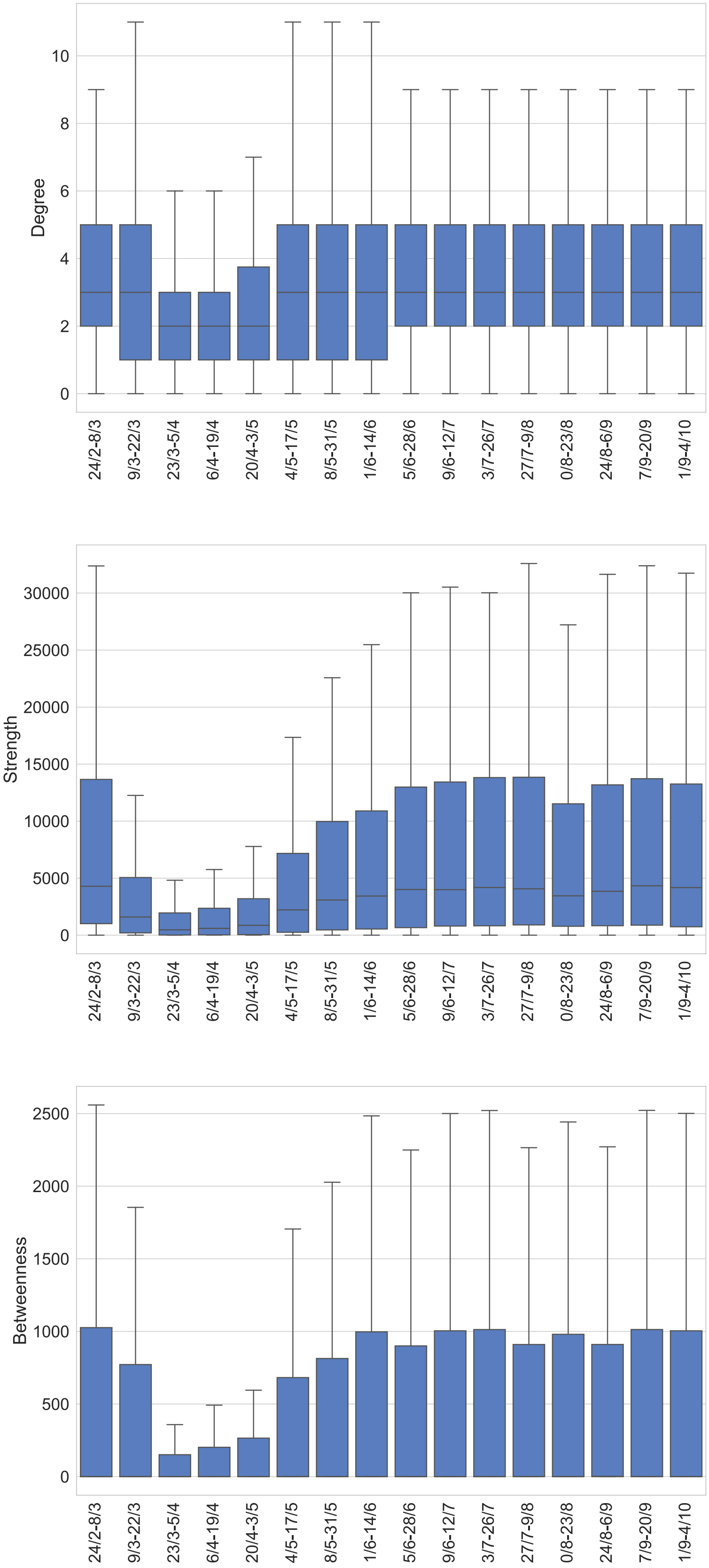

Figure 4. Evolution of distribution of degree, strength and betweenness node centralities.

3 Identifying the Most and Least affected LLMs

In this section, we provide additional results for our analysis on the most and the least affected LLMs. In Figure 5 we show the totality of LLMs for which we have socioeconomic variables in our data. In Figure 6 we show the most and least affected LLMs using $K = 30\%$ and *recovery* variable, as well as the temporal evolution of their variation in nodal efficiency w.r.t baseline (23/2-8/3).

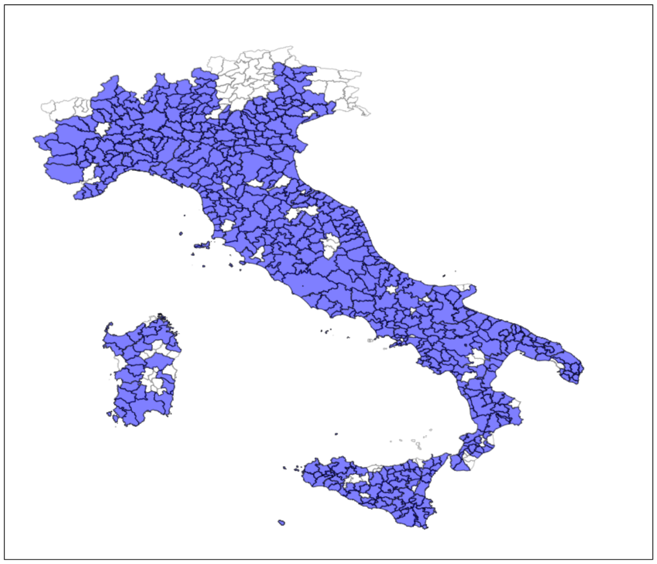

Figure 5. Subdivision of Italy in Local Labor Systems (LLMs). Blue areas correspond to LLMs for which socioeconomic variables are also present in our data.

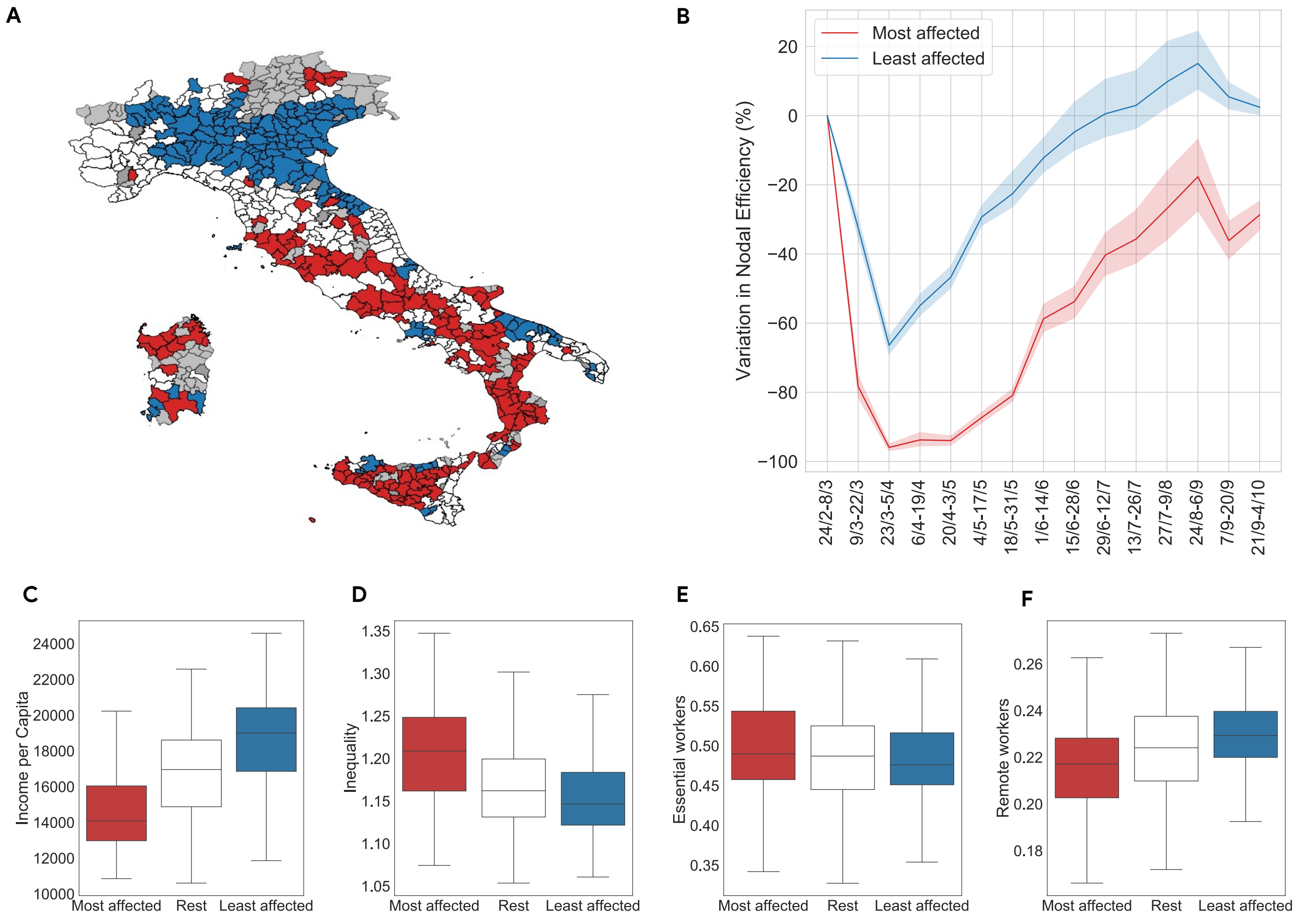

Figure 6. **A.** Most (blue) and least (red) affected LLMs identified using *recovery* variable and $K = 30\%$. Grey color correspond to remaining 40% LLMs in the data. **B.** Temporal evolution of variation in nodal efficiency, w.r.t baseline window of 14 d (23/2-8/3), for most (blue) and least (red) affected LLMs, identified using *recovery* variable and $K = 30\%$.

3.1 Robustness checks for labeling criteria

In this section we provide illustrative examples and additional robustness checks w.r.t the main text for what concerns our definition of the most and least affected LLMs.

In Figure 7 we show the distribution of *lockdown* and *recovery* variables computed averaging over 1 or 2 months of observations, i.e., respectively by computing the mean variation in nodal efficiency of the first two/four windows of 14 d during lockdown/recovery periods w.r.t baseline (23/2-8/3).

In Figure 8 and 9 we show the correspondence of $K\%$ bottom (top) LLMs as identified in *lockdown* and *recovery* distributions, computed respectively over 1 and 2 months of observations.

In Figures 10 and 11 we show the distribution of variation in nodal efficiency for the most and least affected LLMs, identified respectively with *lockdown* and *recovery* variables and with $K = 30\%$, for several windows of 14 d. Distributions are statistically different in all cases (Kolmogorov-Smirnov PVAL $= 0$).

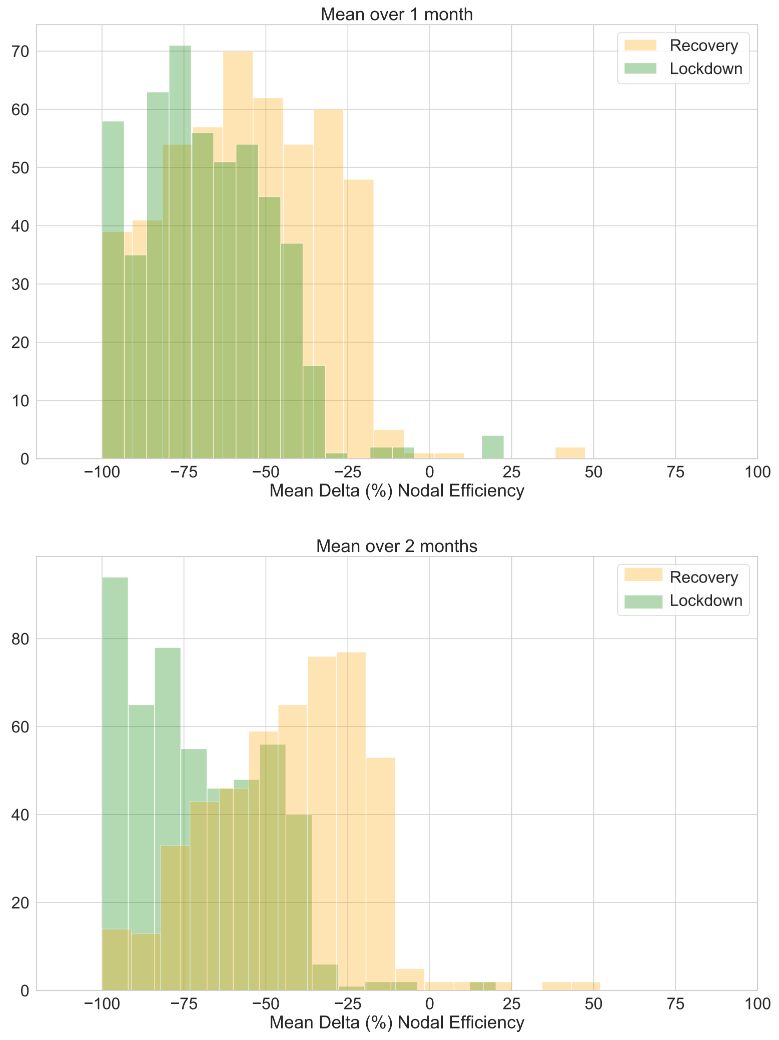

Figure 7. Distribution of values for mean variation in nodal efficiency over two windows (1 month, top) and four windows (2 months, bottom) for both lockdown and recovery periods.

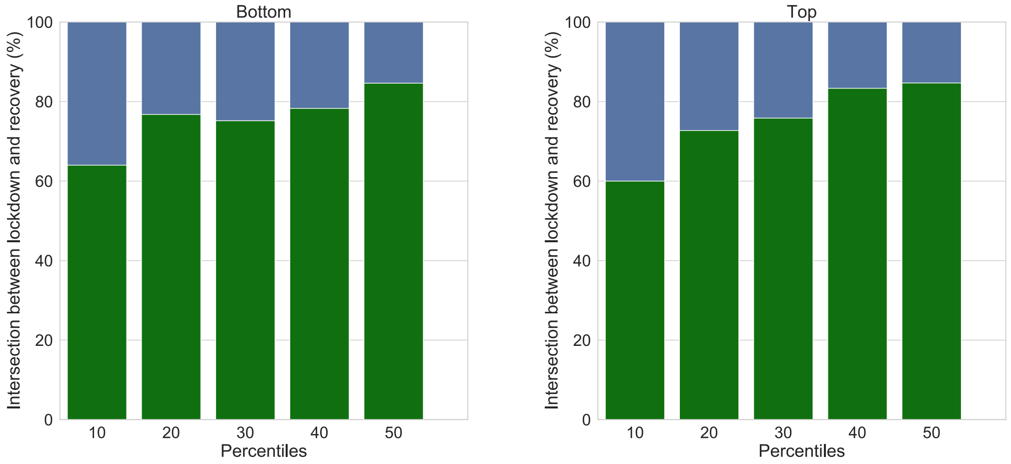

Figure 8. Correspondence of bottom (left) and top (right) LLMs identified in *lockdown* and *recovery* distributions (computed over 1 month of observation), with different percentiles.

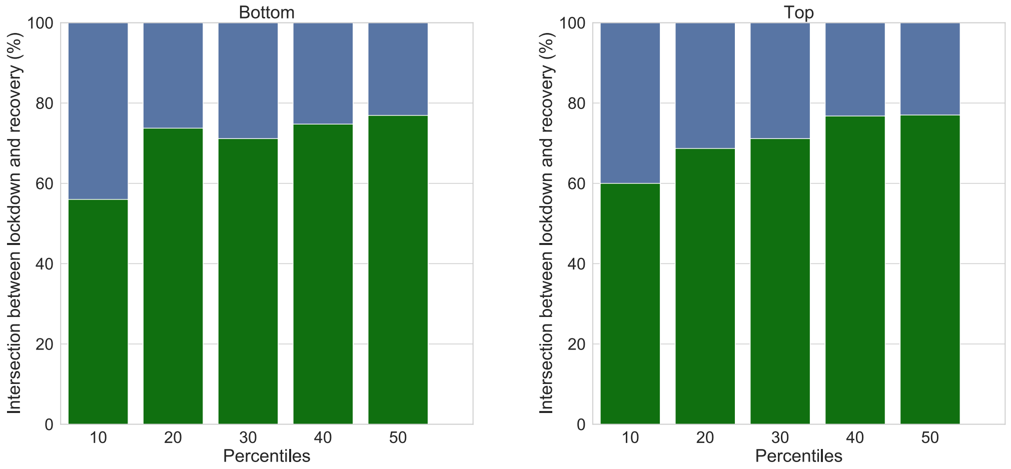

Figure 9. Correspondence of bottom (left) and top (right) LLMs identified in *lockdown* and *recovery* distributions (computed over 2 months of observation), with different percentiles.

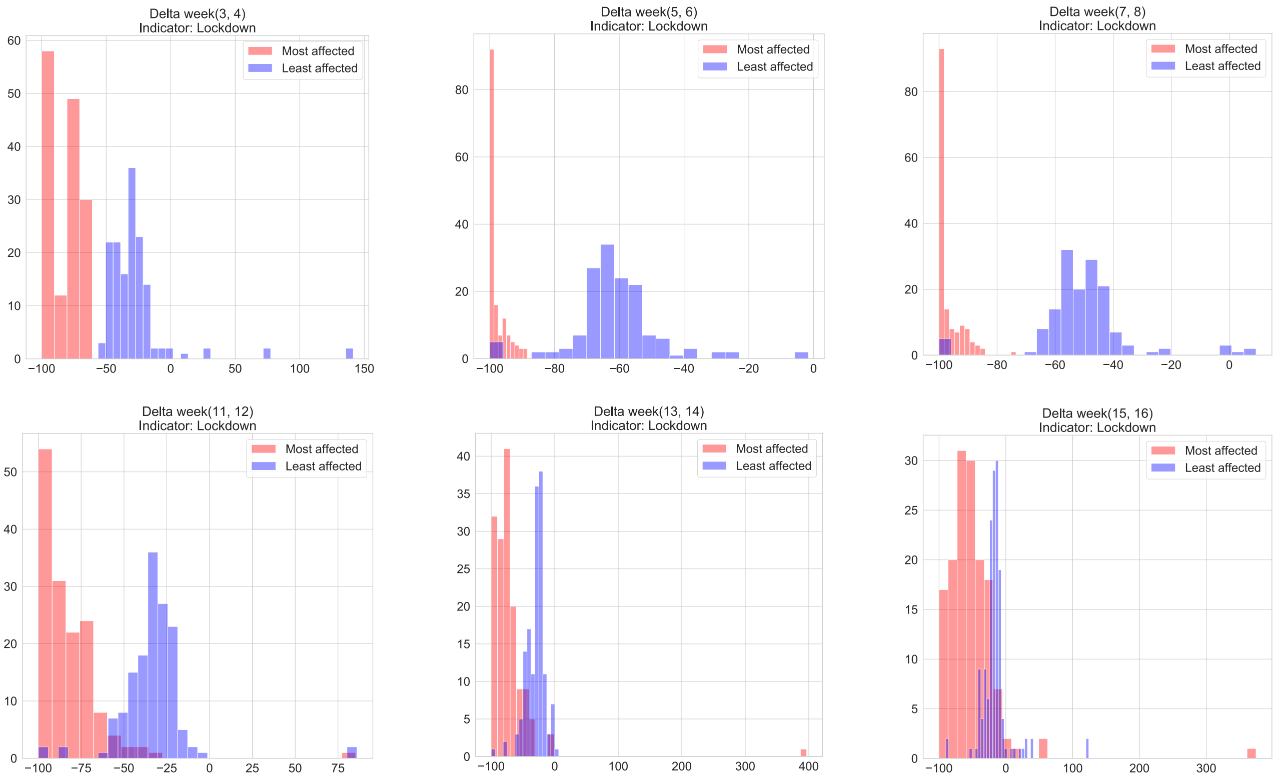

Figure 10. Evolution of distribution of variation of nodal efficiency for the most (red) and least (blue) affected LLMs identified using *crisis* variable (and $K = 30\%$).

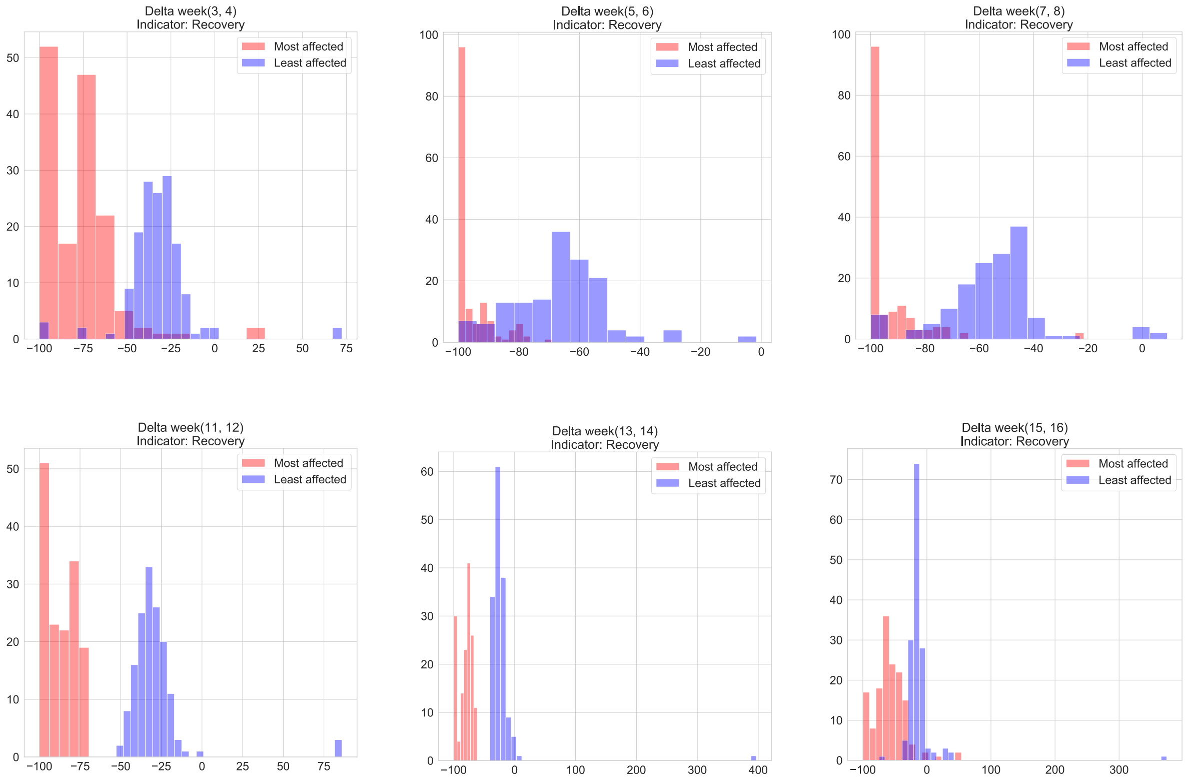

Figure 11. Evolution of distribution of variation of nodal efficiency for the most (red) and least (blue) affected LLMs identified using *recovery* variable (and $K = 30\%$).

3.2 Clustering

In this section, we show results for hierarchical clustering of the most and least affected LLMs evaluated on both *lockdown* and *recovery* criteria with $K = 20\%$ (see Figure 12).

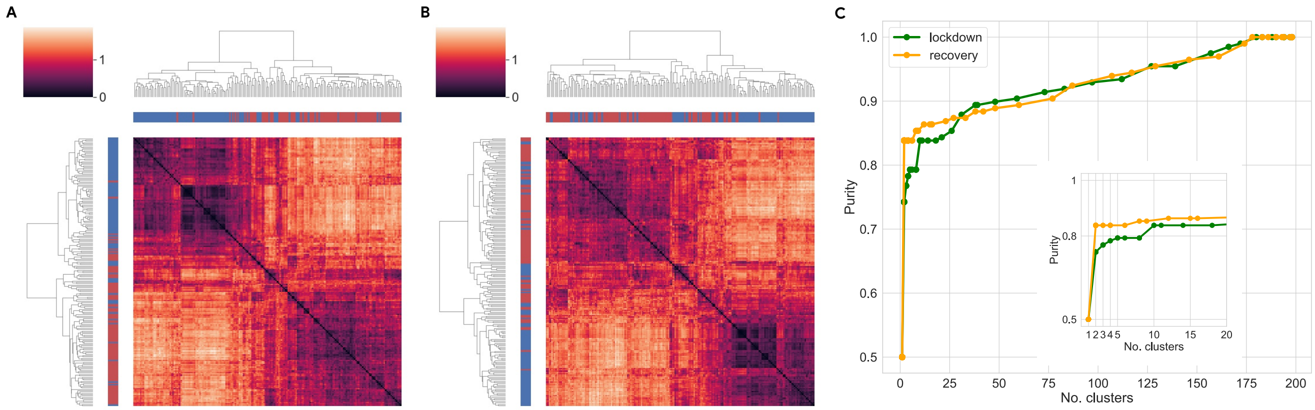

Figure 12. **A-B.** Results of hierarchical clustering using features of the most and least affected LLMs identified with *lockdown* (A) and *recovery* (B) criteria, with $K = 20\%$. Red and blue labels of rows and columns identify respectively the most and least affected areas. **C.** Plot of purity score vs number of clusters for lockdown (green) and recovery (orange) dendrograms, shown respectively for panels A and B.

3.3 Classification

In this section we provide additional results for the classification of the most vs least affected LLMs using socioeconomic variables. In Tables 2 and 3 we show the Area Under the Receiver operating Characteristic curve (AUROC) of several classifiers in the task of binary classifying the most vs least affected LLMs identified respectively with *lockdown* and *recovery* variables (computed over 1 month to define the most and least affected LLMs), and for several values of K.

In Figure 13 we show AUROC values for Random Forest classifier evaluated when considering *lockdown* and *recovery* variables computed either over 1 month (2 windows of 14 d) or 2 months (4 windows of 14 d) for defining the most and least affected LLMs.

**Lockdown**	K=10%	K=20%	K=30%	K=40%	K=50%
Logistic Regression	0.92 (sd 0.06)	0.94 (sd 0.04)	0.89 (sd 0.05)	0.86 (sd 0.03)	0.80 (sd 0.03)
Random Forest	0.90 (sd 0.06)	0.96 (sd 0.03)	0.92 (sd 0.04)	0.87 (sd 0.04)	0.81 (sd 0.02)
SVC Linear	0.88 (sd 0.05)	0.91 (sd 0.04)	0.87 (sd 0.05)	0.85 (sd 0.04)	0.79 (sd 0.02)
K-NN (N=3)	0.90 (sd 0.08)	0.90 (sd 0.05)	0.80 (sd 0.05)	0.79 (sd 0.04)	0.73 (sd 0.05)
K-NN (N=5)	0.88 (sd 0.06)	0.90 (sd 0.04)	0.81 (sd 0.06)	0.82 (sd 0.05)	0.74 (sd 0.04)

Table 2. Area under the Receiver operating Characteristic curve (AUROC) evaluated for several classifiers in the task of classifying the most vs least affected LLMs using *lockdown* criterion.

**Recovery**	K=10%	K=20%	K=30%	K=40%	K=50%
Logistic Regression	0.90 (sd 0.07)	0.94 (sd 0.01)	0.93 (sd 0.03)	0.91 (sd 0.04)	0.82 (sd 0.04)
Random Forest	0.94 (sd 0.04)	0.96 (sd 0.02)	0.95 (sd 0.03)	0.93 (sd 0.03)	0.88 (sd 0.03)
SVC Linear	0.89 (sd 0.08)	0.94 (sd 0.02)	0.93 (sd 0.03)	0.88 (sd 0.03)	0.82 (sd 0.04)
K-NN (N=3)	0.86 (sd 0.09)	0.91 (sd 0.04)	0.89 (sd 0.04)	0.87 (sd 0.04)	0.80 (sd 0.04)
K-NN (N=5)	0.88 (sd 0.08)	0.93 (sd 0.04)	0.90 (sd 0.04)	0.88 (sd 0.04)	0.82 (sd 0.04)

Table 3. Area under the Receiver operating Characteristic curve (AUROC) evaluated for several classifiers in the task of classifying the most vs least affected LLMs using *recovery* criterion and different values of K.

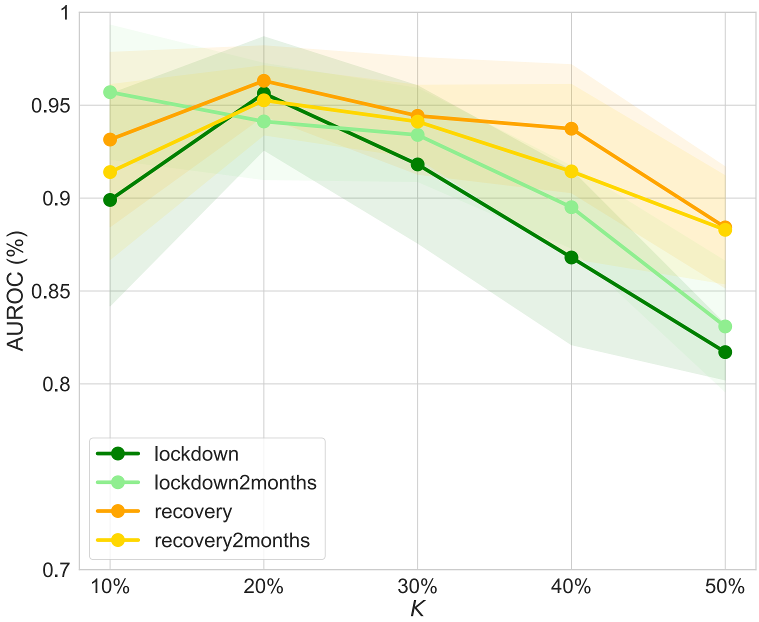

Figure 13. Area Under the Receiving Operator characteristic Curve (AUROC) for Random Forest classifier in the task of classifying the most vs least affected LLMs, identified using different values of K and different criteria, i.e., *lockdown* and *recovery* variables computed either over 1 month or over 2 months.

3.4 Class comparison

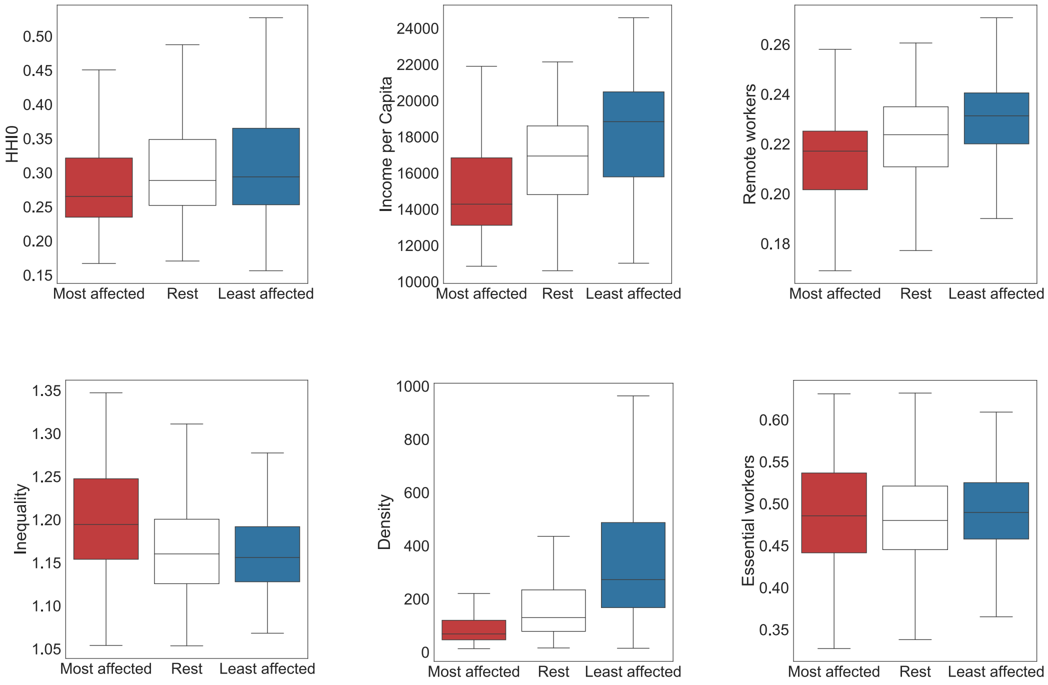

Figure 14. Boxplots for several socioeconomic variables, separating most and least affected LLMs (and the rest of territories), using *lockdown* variable and $K = 30\%$ to identify them.

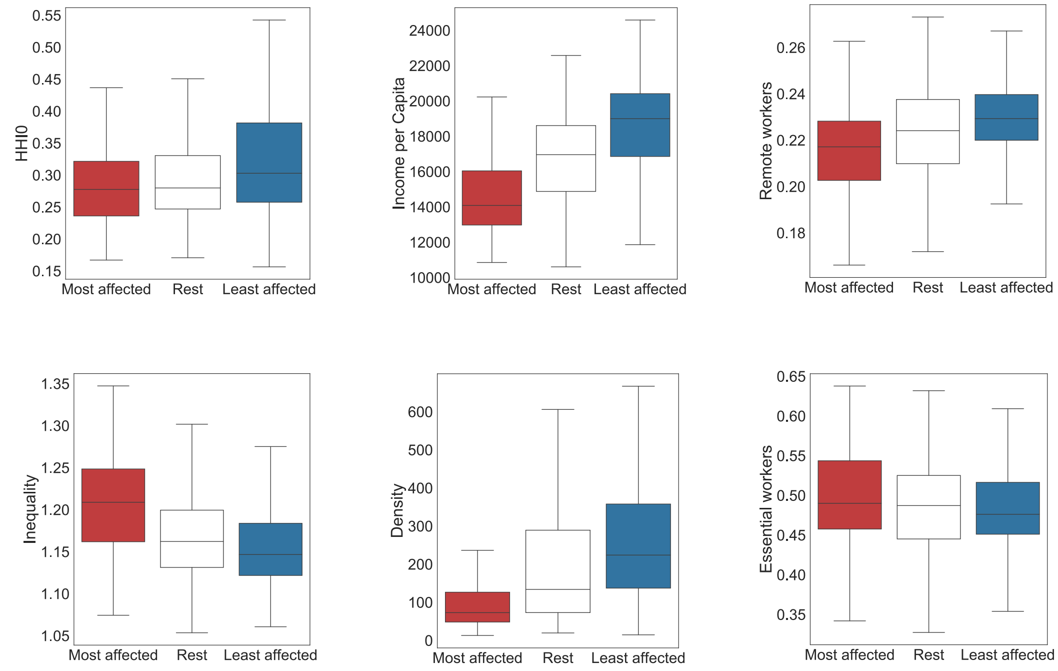

Figure 15. Boxplots for several socioeconomic variables, separating most and least affected LLMs, and remaining ones, using *recovery* variable and $K = 30\%$ to identify them.

3.5 Multiple comparison tests

**Lockdown** ($K = 30\%$)	All	Most affected vs Least affected	Most affected vs Rest	Rest vs Least affected
Income per Capita	0.0000 ***	0.0000 ***	0.0000 ***	0.0002 ***
Population Density	0.0000 ***	0.0000 ***	0.0000 ***	0.0000 ***
Inequality	0.0000 ***	0.0000 ***	0.0000 ***	0.4203
B	0.0020 **	0.0025 **	0.0216 *	0.5724
C	0.0000 ***	0.0000 ***	0.0000 ***	0.0277 *
D	0.0000 ***	0.0000 ***	0.0005 ***	0.0001 ***
E	0.0000 ***	0.0000 ***	0.0000 ***	0.0021 **
F	0.0000 ***	0.0000 ***	0.0000 ***	0.0001 ***
G	0.0000 ***	0.0000 ***	0.0000 ***	0.0000 ***
H	0.0000 ***	0.0000 ***	0.0000 ***	0.0000 ***
I	0.7596	1.0000	1.0000	1.0000
J	0.0000 ***	0.0000 ***	0.0129 *	0.0335 *
L	0.0000 ***	0.0000 ***	0.0000 ***	0.0000 ***
M	0.0012 **	0.0020 **	1.0000	0.0029 **
N	0.0000 ***	0.0000 ***	0.0000 ***	0.1037
P	0.0003 ***	0.0021 **	0.0007 ***	1.0000
Q	0.0000 ***	0.0000 ***	0.0587	0.0000 ***
R	0.0000 ***	0.0000 ***	0.0000 ***	0.0000 ***
S	0.0001 ***	0.0029 **	0.0001 ***	1.0000
HHI0	0.0012 **	0.0046 **	0.0040 **	1.0000
Essential workers	0.8754	1.0000	1.0000	1.0000
Remote workers	0.0000 ***	0.0000 ***	0.0000 ***	0.0442 *
B (%)	0.2655	0.5886	1.0000	0.3335
C (%)	0.0000 ***	0.0000 ***	0.0000 ***	0.0450 *
D (%)	0.0283 *	0.0235 *	0.8287	0.1519
E (%)	0.0010 **	0.0009 ***	0.9427	0.0064 **
F (%)	0.0000 ***	0.0000 ***	0.0000 ***	0.0001 ***
G (%)	0.0002 ***	0.0001 ***	0.0505	0.0503
H (%)	0.0982	0.4365	0.1063	1.0000
I (%)	0.0000 ***	0.0000 ***	0.0000 ***	0.0016 **
J (%)	0.0001 ***	0.0000 ***	0.0565	0.0215 *
L (%)	0.1105	0.7664	0.1083	1.0000
M (%)	0.0011 **	0.0119 *	0.0016 **	1.0000
N (%)	0.0352 *	0.0296 *	0.4161	0.3797
P (%)	0.1635	1.0000	0.3140	0.4236
Q (%)	0.0008 ***	0.0005 ***	0.0725	0.0947
R (%)	0.5511	0.9924	1.0000	0.9400
S (%)	0.0105 *	0.0077 **	0.5583	0.0958

Table 4. Application of Kruskal Wallis test with Conover procedure and Bonferroni pvalue correction to distributions of socioeconomic variables, for the most and least affected LLMs (and rest of them) identified with *lockdown* variable and $K = 30\%$. *, ** and *** indicate respectively significance at $\alpha = 0.05, 0.01, 0.001$.

**Recovery** ($K = 30\%$)	All	Most affected vs Least affected	Most affected vs Rest	Rest vs Least affected
Income per Capita	0.0000 ***	0.0000 ***	0.0000 ***	0.0000 ***
Population Density	0.0000 ***	0.0000 ***	0.0000 ***	0.0000 ***
Inequality	0.0000 ***	0.0000 ***	0.0000 ***	0.0463 *
B	0.0000 ***	0.0000 ***	0.1261	0.0025 **
C	0.0000 ***	0.0000 ***	0.0000 ***	0.0000 ***
D	0.0000 ***	0.0000 ***	0.0000 ***	0.0000 ***
E	0.0000 ***	0.0000 ***	0.0000 ***	0.0000 ***
F	0.0000 ***	0.0000 ***	0.0000 ***	0.0000 ***
G	0.0000 ***	0.0000 ***	0.0000 ***	0.0000 ***
H	0.0000 ***	0.0000 ***	0.0000 ***	0.0000 ***
I	0.0114 *	0.0087 **	0.6989	0.0804
J	0.0000 ***	0.0000 ***	0.0028 **	0.0003 ***
L	0.0000 ***	0.0000 ***	0.0000 ***	0.0000 ***
M	0.0000 ***	0.0000 ***	0.2787	0.0000 ***
N	0.0000 ***	0.0000 ***	0.0000 ***	0.0774
P	0.0000 ***	0.0000 ***	0.0000 ***	0.0011 **
Q	0.0000 ***	0.0000 ***	0.0004 ***	0.0000 ***
R	0.0000 ***	0.0000 ***	0.0000 ***	0.0000 ***
S	0.0000 ***	0.0000 ***	0.0000 ***	0.0001 ***
HHI0	0.0008 ***	0.0004 ***	0.2741	0.0219 *
Essential workers	0.1181	0.3468	0.1547	1.0000
Remote workers	0.0000 ***	0.0000 ***	0.0001 ***	0.0134 *
B (%)	0.1782	0.1932	1.0000	0.5502
C (%)	0.0000 ***	0.0000 ***	0.0000 ***	0.0000 ***
D (%)	0.0031 **	0.0020 **	0.1846	0.1084
E (%)	0.0000 ***	0.0000 ***	0.0676	0.0002 ***
F (%)	0.0000 ***	0.0000 ***	0.0000 ***	0.0000 ***
G (%)	0.0000 ***	0.0000 ***	0.0000 ***	0.0000 ***
H (%)	0.0001 ***	0.0001 ***	0.0066 **	0.2197
I (%)	0.0000 ***	0.0000 ***	0.0011 **	0.0000 ***
J (%)	0.0015 **	0.0013 **	0.0355 *	0.2901
L (%)	0.0144 *	0.2347	0.0113 *	1.0000
M (%)	0.0148 *	0.0372 *	0.0332 *	1.0000
N (%)	0.0222 *	0.0256 *	1.0000	0.0534
P (%)	0.0948	0.0983	0.4771	0.8108
Q (%)	0.0000 ***	0.0000 ***	0.0000 ***	0.0005 ***
R (%)	0.0015 **	0.1307	0.3116	0.0010 ***
S (%)	0.0000 ***	0.0000 ***	1.0000	0.0000 ***

Table 5. Application of Kruskal Wallis test with Conover procedure and Bonferroni pvalue correction to distributions of socioeconomic variables, for the most and least affected LLMs (and rest of them) identified with *recovery* variable and $K = 30\%$. *, ** and *** indicate respectively significance at $\alpha = 0.05, 0.01, 0.001$.

4 Exploratory analysis of the interplay between human mobility and epidemiological variables

In this section we investigate the relationship between human mobility and epidemiological variables. In particular, we illustrate that the most affected administrative units have not been more severely hit by the pandemic and consequently we prove that our results are consistent and not driven by epidemiological variables which we do not include in our analysis. In particular, we focus on the variable extra mortality, which is disclosed by ISTAT and compares the monthly mortality rates of Italian municipalities for the period January - August 2020 with the average value of the correspondent months for the time-frame 2015-2019. As we are concerned with the impact of COVID-19 on human mobility we focus only on the sub-period March-August.

In Figure 16 we show that, contrarily to what might be suspected, the most affected areas in terms of mobility reduction during the *lockdown* are characterized by a lower extra mortality rate in the months of March and April, while administrative units displaying the best mobility performances are those experiencing the highest pandemic intensity. The differences become less evident over the months of May, June, July and August, due to a reduction in the pathogenecity of the virus. However, we confirm that the most and least affected LLMs are not those subject respectively to higher and lower mortality rates, suggesting that our results are not significantly affected by the pandemic intensity.

In Figure 17 we repeat the same analysis on the most and least affected administrative units, identified according to the mobility performances during the *recovery* period. We obtain similar results as LLMs with better mobility performances show higher extra mortality rates in the months of March and April, while differences become less evident in the following months.

In Figure 18 and 19 we perform multiple pairwise comparison tests among the three identified classes (Most affected, Rest and Least affected) to assess whether there is statistical differences among the mortality rates of the three classes over the analysed period.

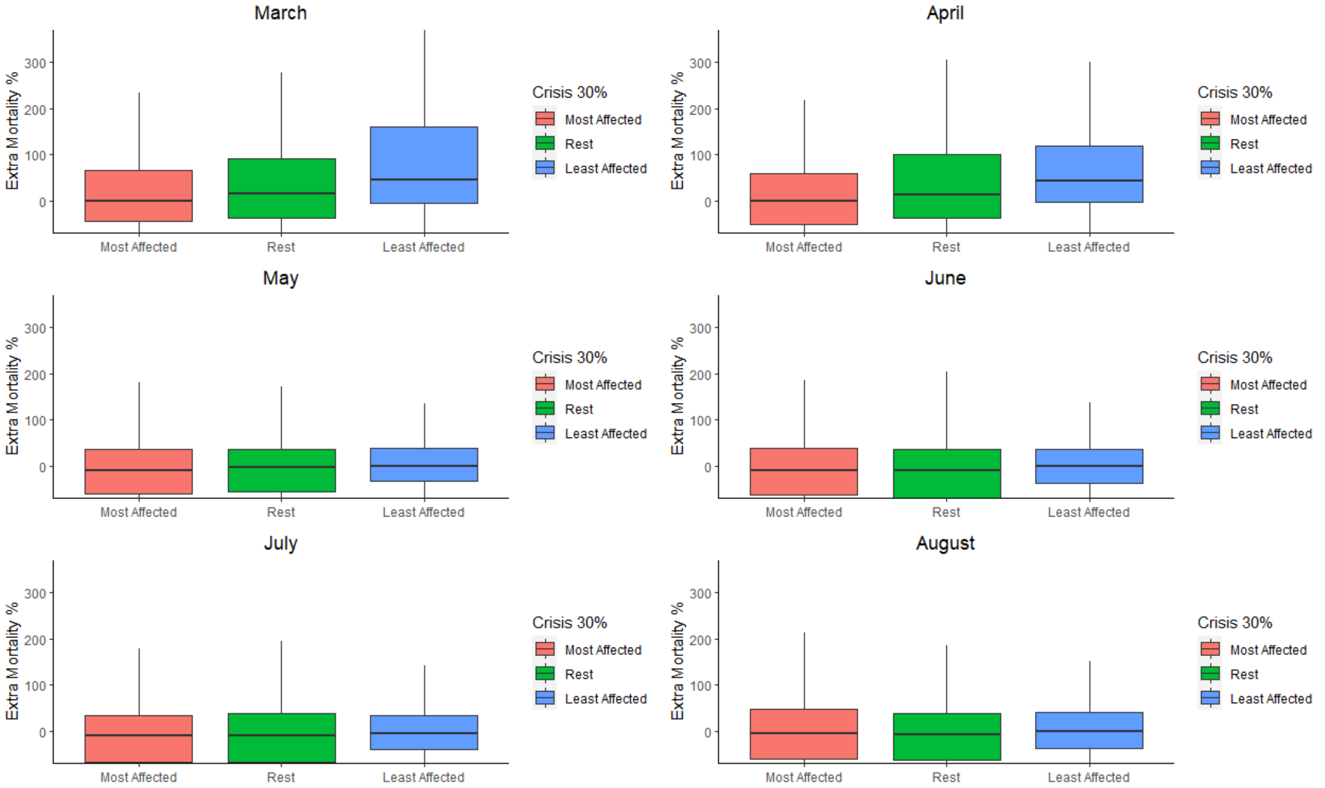

Figure 16. Distribution of Extra Mortality Rate from March 2020 to August 2020 among the Most Affected, Rest and Least Affected LLMs during the lockdown.

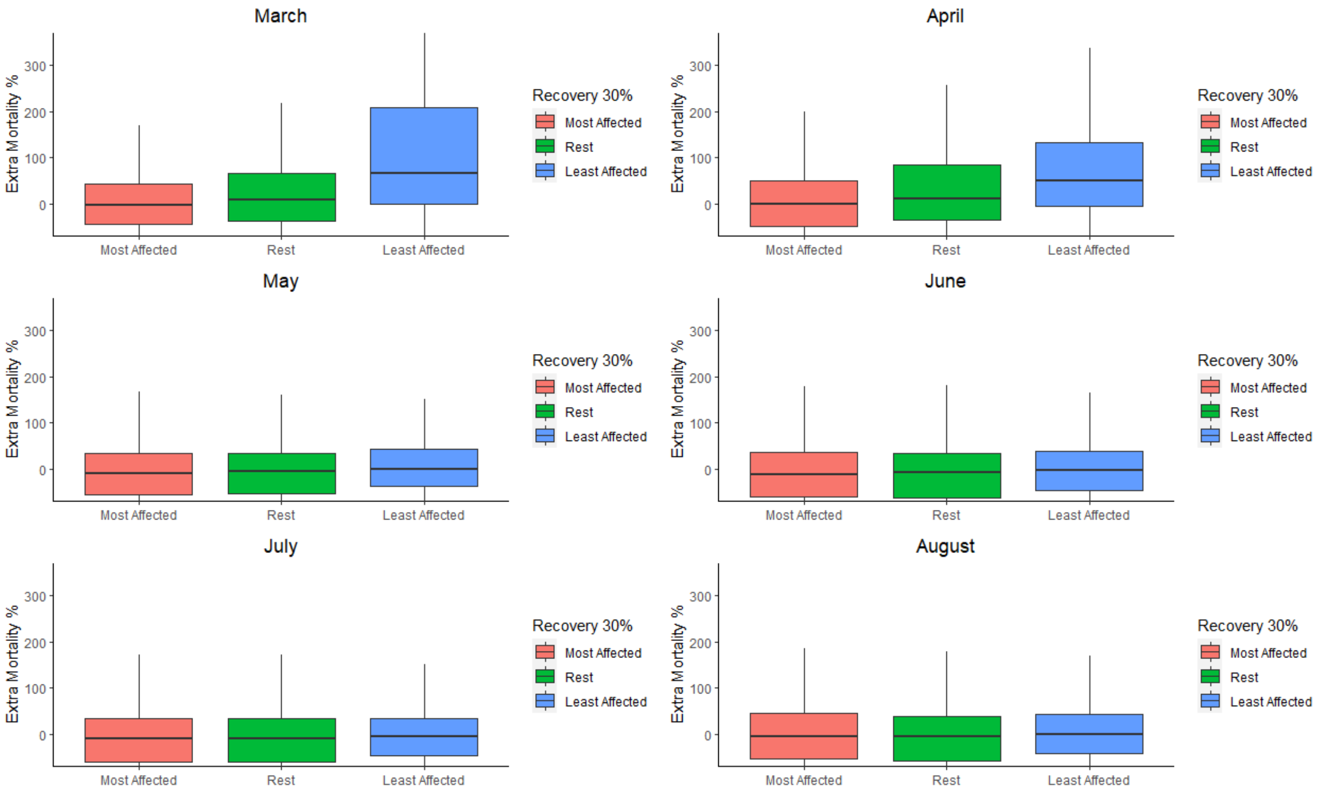

Figure 17. Distribution of Extra Mortality Rate from March 2020 to August 2020 among the Most Affected, Rest and Least Affected LLMs during the recovery.

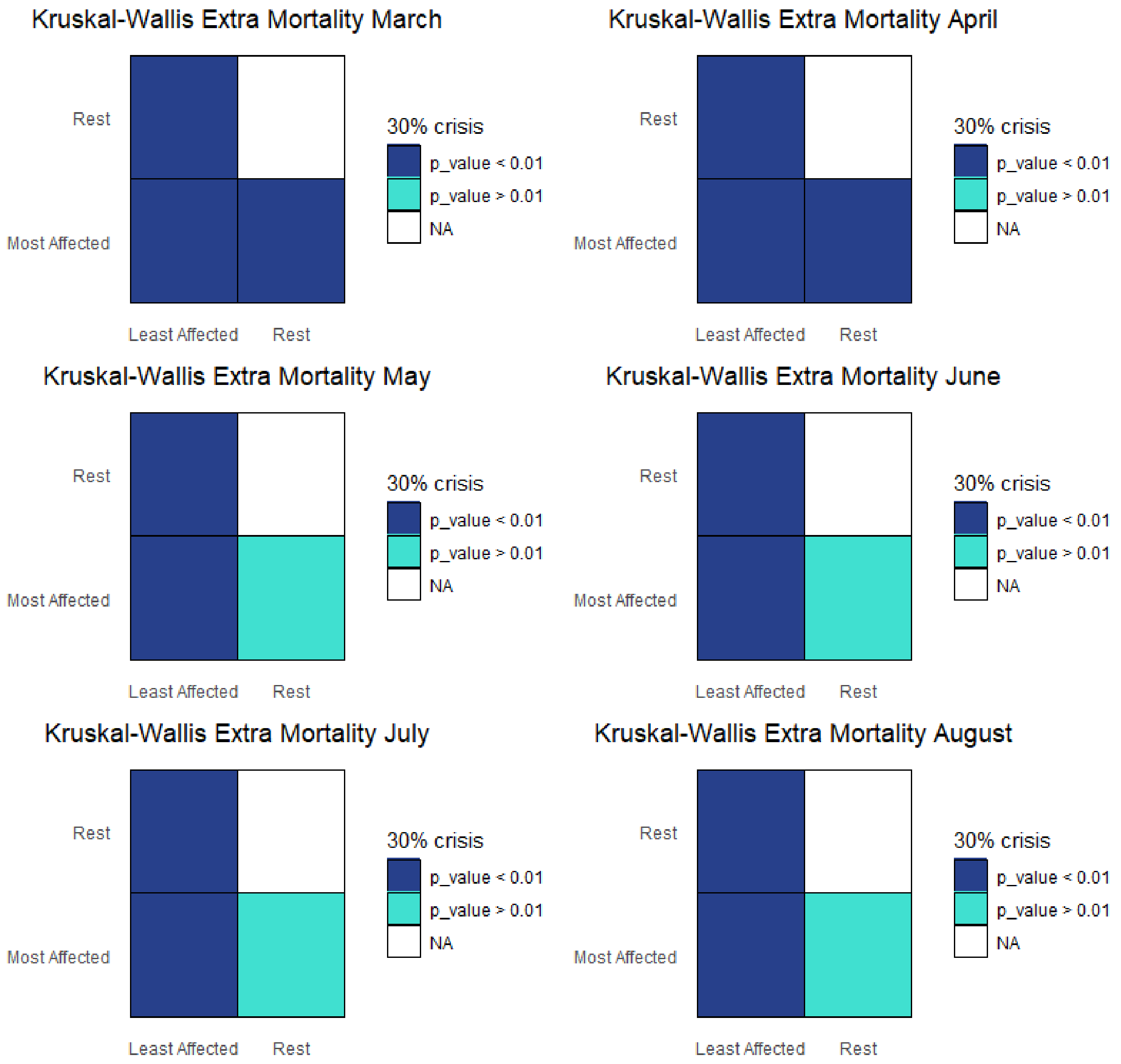

Figure 18. Multiple pair-wise test for Extra Mortality variable, for the Most Affected vs Rest vs Least Affected during the lockdown.

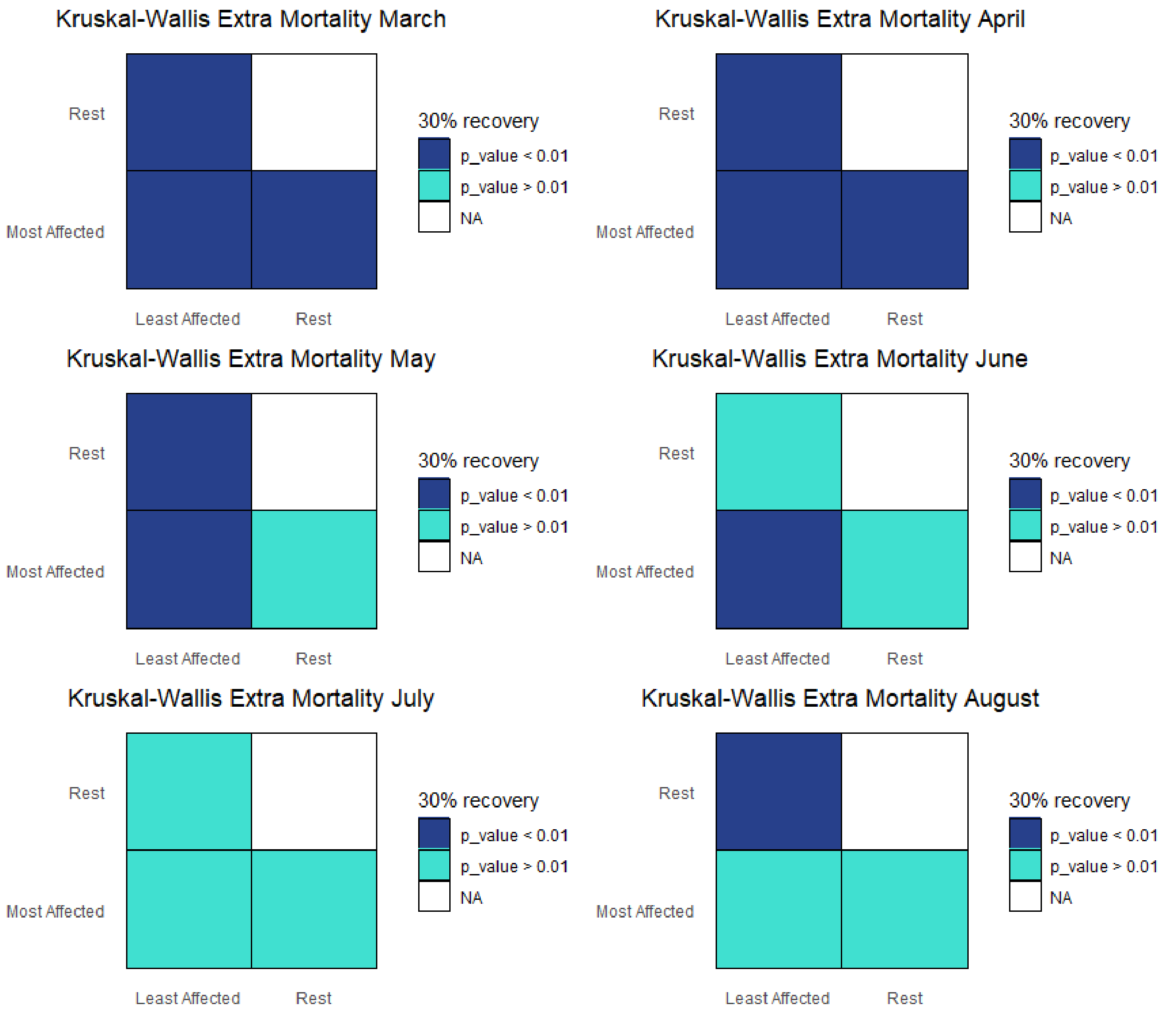

Figure 19. Multiple pair-wise test for Extra Mortality variable, for the Most Affected vs Rest vs Least Affected during the lockdown.

5 Additional codes

Code	Description
A	Agriculture, forestry and fishing
B	Mining and quarrying
C	Manufacturing
D	Electricity, gas, steam and air conditioning supply
E	Water supply, sewerage, waste management and remediation activities
F	Construction
G	Wholesale and retail trade, repair of motor vehicles and motorcycles
H	Transportation and storage
I	Accommodation and food service activities
J	Information and communication activities
K	Financial and insurance activities
L	Real estate activities
M	Professional, scientific and technical activities
N	Administrative and support service activities
O	Public administration and defence, compulsory social security
P	Education
Q	Human health and social work activities
R	Arts, entertainment and recreation
S	Other service activities
T	Activities of households as employers, undifferentiated goods - and services-producing activities of households for own use
U	Activities of extraterritorial organizations and bodies

Table 6. NACE Rev. 2/ATECO Codes

ITC	North-West (NW)	ITC1	Piemonte
		ITC2	Valle d'Aosta/Vallée d'Aoste
		ITC3	Liguria
		ITC4	Lombardia
ITH	North-East (NE)	ITH1	Provincia Autonoma di Bolzano/Bozen
		ITH2	Provincia Autonoma di Trento
		ITH3	Veneto
		ITH4	Friuli-Venezia Giulia
		ITH5	Emilia-Romagna
ITI	Center (CEN)	ITI1	Toscana
		ITI2	Umbria
		ITI3	Marche
		ITI4	Lazio
ITF	South (SOU)	ITF1	Abruzzo
		ITF2	Molise
		ITF3	Campania
		ITF4	Puglia
		ITF5	Basilicata
		ITF6	Calabria
ITG	Insular Italy (ISL)	ITG1	Sicilia
		ITG2	Sardegna

Table 7. Correspondence Italian macroareas - NUTS Codes

6 Quantile regression results

In Tables 8 and 9 we report full results for our quantile regression models. To identify sectors and territories we use the coding reported in Tables 6 and 7.

In Table 10 and 11 we report robustness results for quantile regressions with heteroskedastic robust standard errors obtained via wild bootstrap[3] with 1000 iterations. We can see that in general standard errors increase, resulting in a decrease of statistical significance, however the majority of our results hold with only few effects which are no longer significant. In particular, during recovery, we no longer see an effect from Essential Workers variable and the Administrative and Education sectors (N and P) are no longer significant among less affected units.

Next, we briefly add some further comments on our results, which we left out from the main text for length reasons.

Productive structure of LLMs. First, we highlight the effect of Transportation and Accommodation sectors, which are particularly interesting since they both represent mobility-dependent sectors which have been exempted from restrictions during lockdown. We observe that they are strategic for recovery among the most affected areas, i.e. higher levels of revenue per employee in these sectors are associated with faster rates of recovery, and detrimental for the least affected areas where the effect is reversed. Moreover, we find that these effects were in part present also during lockdown: among the most affected areas for the Transportation sector, and among the least affected ones for the Accommodation sector. Our interpretation is that firms in the Transportation sector exploited the exemption from lockdown measures and avoided higher losses in the most affected areas, when highly present in a specific LLM. Firms in the Accommodation sector, on the contrary, being constrained by customer movements, were able to benefit only in the most affected areas during the recovery, while slowing down least affected ones in both periods.

Next, we observe that for two specific sectors, Administrative Services and Education, higher values of revenue per employee are associated with increasing reduction in mobility during lockdown among the least affected areas (and also among the most affected areas for the Education sector). This can be explained by a shift from work in-person to remote work, which has regarded especially the Education sector. However, the shift is not permanent, since during the recovery the effect has reverted for the Administrative Services sector and is not present for the Education sector (to be precise it is significant only at the extreme tail of the distribution).

Finally, we highlight the peculiar behavior of the sector Real Estate Activities, which exhibits a positive and significant coefficient for all quantiles in both periods. A probable explanation for this is that it is associated with densely populated areas, which show a similar pattern.

Territorial controls. First, for the Construction sector we find a negative coefficient, during lockdown, for the most affected LLMs in the NE area and the least affected ones in the insular area of Italy (ISL). Moreover, during recovery, we find a negative and significant coefficient of the main variable for all quantiles, signaling a persistent negative effect of the sector on recovery rates. This suggests that the Construction sector might be one of the most affected by restrictions, most likely because it is less suitable for remote work.

Second, all other main sector effects are not significant when interacted with macroarea dummies. Moreover, interaction effects are usually not significant except for limited cases.

6.1 Results with default standard errors

Table 8. Quantile regression model with macroarea interactions

	Dependent variable: lockdown indicator						
	q10	q20	q30	q70	q80	q90	OLS
Income per capita	0.303***	0.234***	0.167**	0.243**	0.243***	0.169**	0.208**
	(0.077)	(0.069)	(0.078)	(0.097)	(0.064)	(0.074)	(0.090)
Population density_2016	0.155***	0.136***	0.147***	0.226***	0.146***	0.109**	0.179***
	(0.045)	(0.040)	(0.046)	(0.057)	(0.037)	(0.043)	(0.053)
Income Inequality	0.019	−0.054	−0.046	−0.071	−0.005	0.055	−0.059
	(0.041)	(0.036)	(0.041)	(0.051)	(0.033)	(0.039)	(0.047)
Revenue concentration	0.022	0.064	0.095**	0.043	−0.007	0.033	0.051
	(0.045)	(0.040)	(0.046)	(0.057)	(0.037)	(0.043)	(0.053)
Essential workers	−0.011	−0.016	0.025	−0.003	−0.069	−0.018	−0.076
	(0.061)	(0.054)	(0.061)	(0.076)	(0.050)	(0.058)	(0.071)
Remote workers	0.134**	0.096	0.106	0.036	0.092*	0.004	0.069
	(0.068)	(0.060)	(0.068)	(0.085)	(0.056)	(0.064)	(0.079)
Revenues per employee B	0.029	0.068	0.036	−0.030	−0.041	−0.037	−0.009
	(0.048)	(0.043)	(0.049)	(0.061)	(0.040)	(0.046)	(0.057)
Revenues per employee C	0.035	0.064	0.112	0.073	0.078	0.001	0.166
	(0.185)	(0.165)	(0.186)	(0.232)	(0.153)	(0.176)	(0.216)
Revenues per employee D	0.107**	0.071*	0.026	−0.026	−0.006	−0.004	−0.020
	(0.047)	(0.041)	(0.047)	(0.058)	(0.038)	(0.044)	(0.054)
Revenues per employee E	0.031	0.026	−0.004	0.076	0.023	−0.014	0.019
	(0.058)	(0.052)	(0.058)	(0.073)	(0.048)	(0.055)	(0.068)
Revenues per employee F	0.009	0.028	−0.024	−0.033	−0.001	−0.388**	−0.131
	(0.166)	(0.148)	(0.167)	(0.208)	(0.137)	(0.158)	(0.194)
Revenues per employee G	0.027	0.100*	0.095	−0.028	0.004	−0.051	0.028
	(0.067)	(0.059)	(0.067)	(0.084)	(0.055)	(0.064)	(0.078)
Revenues per employee H	0.164***	0.131**	0.125**	0.007	0.067	−0.035	0.064
	(0.060)	(0.053)	(0.060)	(0.075)	(0.049)	(0.057)	(0.070)
Revenues per employee I	−0.040	−0.053	−0.095*	−0.136**	−0.140***	−0.116**	−0.082
	(0.049)	(0.043)	(0.049)	(0.061)	(0.040)	(0.046)	(0.057)
Revenues per employee J	−0.056	−0.023	−0.038	−0.028	0.017	−0.062	−0.047
	(0.047)	(0.042)	(0.047)	(0.059)	(0.039)	(0.045)	(0.055)
Revenues per employee L	0.115	0.226***	0.210***	0.162*	0.172***	0.235***	0.167*
	(0.076)	(0.067)	(0.076)	(0.095)	(0.062)	(0.072)	(0.088)
Revenues per employee M	−0.084	−0.132	−0.126	−0.118	−0.141*	−0.044	−0.136
	(0.097)	(0.086)	(0.097)	(0.121)	(0.080)	(0.092)	(0.113)
Revenues per employee N	0.129***	0.090**	0.064	0.003	−0.012	−0.067*	0.044
	(0.042)	(0.038)	(0.043)	(0.053)	(0.035)	(0.040)	(0.050)

Revenues per employee P	−0.174***	−0.085	−0.086	−0.064	−0.101**	−0.025	−0.084
	(0.062)	(0.055)	(0.062)	(0.077)	(0.051)	(0.059)	(0.072)
Revenues per employee Q	−0.139	−0.150*	−0.071	−0.011	−0.077	0.006	−0.075
	(0.096)	(0.085)	(0.096)	(0.120)	(0.079)	(0.091)	(0.112)
Revenues per employee R	−0.016	−0.041	−0.181	−0.117	0.298	0.358	−0.125
	(0.360)	(0.320)	(0.361)	(0.451)	(0.297)	(0.343)	(0.420)
Revenues per employee S	0.003	−0.023	−0.002	−0.096	−0.173***	−0.259***	−0.127
	(0.069)	(0.062)	(0.070)	(0.087)	(0.057)	(0.066)	(0.081)
Employees B	−0.207*	−0.230**	−0.145	−0.006	−0.056	−0.092	−0.182
	(0.122)	(0.108)	(0.122)	(0.153)	(0.100)	(0.116)	(0.142)
Employees C	0.372**	0.414***	0.323**	−0.140	−0.022	0.129	0.221
	(0.149)	(0.133)	(0.150)	(0.187)	(0.123)	(0.142)	(0.174)
Employees D	−0.343	−0.592***	−0.646***	−0.616**	−0.265	−0.128	−0.406
	(0.221)	(0.196)	(0.222)	(0.276)	(0.182)	(0.210)	(0.257)
Employees E	−0.016	0.205	0.150	0.105	−0.242	−0.218	−0.199
	(0.195)	(0.173)	(0.195)	(0.244)	(0.160)	(0.185)	(0.227)
Employees F	−0.021	−0.170	−0.067	0.041	0.323	0.068	−0.024
	(0.325)	(0.289)	(0.326)	(0.407)	(0.268)	(0.309)	(0.379)
Employees G	1.762***	0.628	0.329	1.140	0.696	0.045	0.345
	(0.608)	(0.540)	(0.610)	(0.761)	(0.501)	(0.578)	(0.708)
Employees H	−0.328*	−0.379**	−0.374**	−0.415*	0.045	0.199	−0.238
	(0.184)	(0.163)	(0.184)	(0.230)	(0.151)	(0.175)	(0.214)
Employees I	−1.347***	−0.751***	−0.186	0.044	−0.059	−0.093	−0.442
	(0.304)	(0.270)	(0.306)	(0.381)	(0.251)	(0.290)	(0.355)
Employees J	0.724*	0.440	0.180	0.308	0.003	−0.396	−0.046
	(0.410)	(0.364)	(0.411)	(0.513)	(0.338)	(0.390)	(0.478)
Employees L	1.897***	1.513***	1.233*	0.110	0.011	−0.402	0.505
	(0.626)	(0.557)	(0.629)	(0.785)	(0.516)	(0.596)	(0.730)
Employees M	−2.185***	−1.090**	−0.696	−0.585	−0.385	0.552	−0.382
	(0.578)	(0.513)	(0.580)	(0.724)	(0.476)	(0.550)	(0.674)
Employees N	−0.290	−0.491	−0.523	−0.698	−0.649	−0.131	−0.265
	(0.525)	(0.466)	(0.527)	(0.657)	(0.433)	(0.499)	(0.612)
Employees P	−0.697***	−0.162	−0.075	−0.051	−0.088	−0.147	−0.085
	(0.193)	(0.171)	(0.194)	(0.242)	(0.159)	(0.184)	(0.225)
Employees Q	−0.373	−0.171	−0.070	0.099	0.056	−0.099	−0.056
	(0.295)	(0.262)	(0.296)	(0.370)	(0.243)	(0.281)	(0.344)
Employees R	0.882***	0.731***	0.411**	0.143	0.165	0.327*	0.500**
	(0.208)	(0.184)	(0.208)	(0.260)	(0.171)	(0.198)	(0.242)
Employees S	0.180	0.166	0.236	0.544	0.459	0.344	0.828
	(0.498)	(0.442)	(0.500)	(0.623)	(0.410)	(0.474)	(0.580)

nuts_ITH	0.872***	0.421	0.771***	0.723**	0.486**	0.349	0.550
	(0.289)	(0.257)	(0.291)	(0.362)	(0.239)	(0.275)	(0.337)
nuts_ITI	−0.047	−0.095	−0.139	−0.264	−0.351**	−0.030	−0.227
	(0.199)	(0.177)	(0.200)	(0.249)	(0.164)	(0.189)	(0.232)
nuts_ITF	0.455	0.215	−0.099	0.220	0.386	0.921***	0.154
	(0.285)	(0.253)	(0.286)	(0.356)	(0.235)	(0.271)	(0.332)
nuts_ITG	0.237	0.147	0.071	−0.191	−0.247*	−1.025***	−0.253
	(0.166)	(0.148)	(0.167)	(0.208)	(0.137)	(0.158)	(0.194)
Revenues per employee C:nuts_ITF	−0.0002	−0.225	−0.175	−0.062	−0.225	0.158	−0.197
	(0.268)	(0.238)	(0.269)	(0.335)	(0.221)	(0.255)	(0.312)
Revenues per employee F:nuts_ITF	0.082	−0.135	−0.221	−0.005	0.113	0.485*	−0.155
	(0.286)	(0.254)	(0.287)	(0.359)	(0.236)	(0.272)	(0.334)
Revenues per employee Q:nuts_ITF	0.208	0.324***	0.167	−0.060	0.183	0.263**	0.159
	(0.140)	(0.124)	(0.140)	(0.175)	(0.115)	(0.133)	(0.163)
Revenues per employee R:nuts_ITF	0.095	0.122	0.209	0.367	−0.043	0.154	0.296
	(0.421)	(0.374)	(0.423)	(0.528)	(0.347)	(0.401)	(0.491)
Revenues per employee C:nuts_ITH	0.363	0.388	1.247***	0.571	0.278	0.209	0.582
	(0.426)	(0.379)	(0.428)	(0.534)	(0.351)	(0.406)	(0.497)
Revenues per employee F:nuts_ITH	−0.965***	−0.803***	−0.760***	−0.413	−0.334	−0.120	−0.444
	(0.267)	(0.237)	(0.268)	(0.334)	(0.220)	(0.254)	(0.311)
Revenues per employee Q:nuts_ITH	0.986***	0.938***	0.508***	0.058	0.176	0.083	0.387*
	(0.176)	(0.156)	(0.176)	(0.220)	(0.145)	(0.167)	(0.205)
Revenues per employee R:nuts_ITH	−0.031	−0.167	0.153	0.117	−0.399	−0.461	0.004
	(0.426)	(0.379)	(0.428)	(0.534)	(0.351)	(0.405)	(0.497)
Revenues per employee C:nuts_ITI	−0.235	−0.198	−0.128	0.149	0.293	0.840***	0.020
	(0.275)	(0.245)	(0.276)	(0.345)	(0.227)	(0.262)	(0.321)
Revenues per employee F:nuts_ITI	−0.295	−0.465**	−0.312	0.135	0.117	0.449**	−0.129
	(0.229)	(0.204)	(0.230)	(0.287)	(0.189)	(0.218)	(0.267)
Revenues per employee Q:nuts_ITI	0.106	0.040	−0.023	0.025	0.042	−0.135	0.048
	(0.155)	(0.138)	(0.156)	(0.195)	(0.128)	(0.148)	(0.181)
Revenues per employee R:nuts_ITI	0.103	0.041	0.206	0.123	−0.437	−0.108	0.143
	(0.450)	(0.400)	(0.452)	(0.564)	(0.371)	(0.428)	(0.525)
Revenues per employee C:nuts_ITG	0.079	0.075	0.048	−0.004	−0.011	−0.152*	−0.040
	(0.093)	(0.083)	(0.094)	(0.117)	(0.077)	(0.089)	(0.109)
Revenues per employee F:nuts_ITG	−0.058	−0.099	−0.017	−0.468**	−0.571***	−1.652***	−0.568***
	(0.164)	(0.146)	(0.165)	(0.205)	(0.135)	(0.156)	(0.191)
Revenues per employee Q:nuts_ITG	0.155***	0.160***	0.104*	0.089	0.145***	0.164***	0.185***
	(0.057)	(0.050)	(0.057)	(0.071)	(0.047)	(0.054)	(0.066)
Revenues per employee R:nuts_ITG	0.152	−0.009	0.031	−0.017	−0.252	−0.097	−0.027
	(0.223)	(0.198)	(0.224)	(0.279)	(0.184)	(0.212)	(0.259)

Constant	-0.868^{***}	-0.628^{***}	-0.428^{***}	0.039	0.223^{*}	0.073	-0.269
	(0.160)	(0.142)	(0.161)	(0.200)	(0.132)	(0.152)	(0.186)
AIC	1077.58	1072.87	1108.63	1261.38	1373.51	1453.74	1256.68
BIC	1325.65	1320.94	1356.7	1509.45	1621.58	1701.81	1136.68

Note: *p<0.1; **p<0.05; ***p<0.01

Table 9. Quantile regression model with macroarea interactions

	Dependent variable: recovery indicator						
	q10	q20	q30	q70	q80	q90	OLS
Income per capita	0.226***	0.182***	0.162**	0.217***	0.223***	0.281***	0.217**
	(0.074)	(0.065)	(0.068)	(0.055)	(0.062)	(0.058)	(0.087)
Population density_2016	0.119***	0.101***	0.082**	0.103***	0.118***	0.080**	0.125**
	(0.043)	(0.038)	(0.040)	(0.032)	(0.037)	(0.034)	(0.051)
Income Inequality	0.004	−0.040	−0.086**	−0.107***	−0.110***	−0.090***	−0.079*
	(0.039)	(0.034)	(0.036)	(0.029)	(0.033)	(0.030)	(0.046)
Revenue concentration	0.075*	0.056	0.065	0.029	0.031	−0.031	0.041
	(0.043)	(0.038)	(0.040)	(0.032)	(0.037)	(0.034)	(0.051)
Essential workers	−0.077	−0.107**	−0.026	−0.129***	−0.125**	−0.178***	−0.193***
	(0.058)	(0.051)	(0.053)	(0.043)	(0.049)	(0.045)	(0.068)
Remote workers	0.148**	0.145**	0.090	0.078	0.051	−0.012	0.082
	(0.064)	(0.057)	(0.059)	(0.048)	(0.055)	(0.051)	(0.076)
Revenues per employee B	0.035	0.025	−0.018	0.018	0.020	0.086**	0.066
	(0.046)	(0.041)	(0.043)	(0.034)	(0.039)	(0.036)	(0.054)
Revenues per employee C	0.328*	0.161	−0.007	0.092	−0.075	0.074	0.024
	(0.177)	(0.156)	(0.163)	(0.131)	(0.149)	(0.138)	(0.208)
Revenues per employee D	0.028	0.011	−0.010	−0.037	−0.005	−0.062*	−0.038
	(0.044)	(0.039)	(0.041)	(0.033)	(0.037)	(0.035)	(0.052)
Revenues per employee E	0.084	0.096*	0.152***	−0.013	−0.016	−0.025	0.025
	(0.055)	(0.049)	(0.051)	(0.041)	(0.047)	(0.043)	(0.065)
Revenues per employee F	−0.475***	−0.556***	−0.566***	−0.386***	−0.400***	−0.603***	−0.566***
	(0.158)	(0.140)	(0.146)	(0.117)	(0.134)	(0.124)	(0.186)
Revenues per employee G	−0.168***	−0.046	−0.007	−0.053	−0.100*	−0.208***	−0.065
	(0.064)	(0.056)	(0.059)	(0.047)	(0.054)	(0.050)	(0.075)
Revenues per employee H	0.048	0.134***	0.140***	0.065	0.061	−0.059	0.053
	(0.057)	(0.050)	(0.053)	(0.042)	(0.048)	(0.045)	(0.067)
Revenues per employee I	0.102**	0.085**	0.054	−0.057*	−0.095**	−0.095***	−0.007
	(0.047)	(0.041)	(0.043)	(0.034)	(0.039)	(0.036)	(0.055)
Revenues per employee J	−0.088*	−0.040	−0.050	−0.013	0.026	−0.015	−0.066
	(0.045)	(0.040)	(0.041)	(0.033)	(0.038)	(0.035)	(0.053)
Revenues per employee L	0.257***	0.254***	0.237***	0.182***	0.178***	0.206***	0.206**
	(0.072)	(0.064)	(0.066)	(0.053)	(0.061)	(0.056)	(0.085)
Revenues per employee M	−0.032	−0.023	−0.088	−0.107	−0.036	0.047	−0.165
	(0.092)	(0.081)	(0.085)	(0.068)	(0.078)	(0.072)	(0.108)
Revenues per employee N	0.026	0.049	0.102***	0.053*	0.012	0.026	0.055
	(0.040)	(0.036)	(0.037)	(0.030)	(0.034)	(0.032)	(0.048)
Revenues per employee P	−0.022	−0.012	0.050	−0.024	−0.077	−0.058	−0.041
	(0.059)	(0.052)	(0.054)	(0.044)	(0.050)	(0.046)	(0.069)

Revenues per employee Q	0.179*	0.040	0.049	0.030	−0.019	0.035	0.061
	(0.091)	(0.081)	(0.084)	(0.068)	(0.077)	(0.071)	(0.107)
Revenues per employee R	−0.158	0.163	0.042	0.396	0.523*	−0.013	−0.010
	(0.343)	(0.303)	(0.316)	(0.254)	(0.290)	(0.269)	(0.404)
Revenues per employee S	−0.098	−0.077	−0.015	−0.037	−0.160***	−0.260***	−0.018
	(0.066)	(0.059)	(0.061)	(0.049)	(0.056)	(0.052)	(0.078)
Employees B	−0.042	−0.075	−0.079	−0.083	−0.065	−0.071	−0.164
	(0.116)	(0.103)	(0.107)	(0.086)	(0.098)	(0.091)	(0.137)
Employees C	0.160	0.092	0.104	−0.055	−0.179	−0.195*	−0.019
	(0.142)	(0.126)	(0.131)	(0.105)	(0.120)	(0.112)	(0.168)
Employees D	−0.303	−0.242	−0.415**	−0.321**	−0.189	−0.036	−0.222
	(0.210)	(0.186)	(0.194)	(0.156)	(0.178)	(0.165)	(0.248)
Employees E	0.024	0.002	−0.038	−0.065	−0.165	−0.083	−0.066
	(0.185)	(0.164)	(0.171)	(0.137)	(0.157)	(0.145)	(0.218)
Employees F	0.041	0.155	0.211	0.087	0.294	0.525**	0.209
	(0.309)	(0.274)	(0.285)	(0.229)	(0.262)	(0.242)	(0.364)
Employees G	0.944	0.378	0.046	0.541	0.627	0.571	0.192
	(0.579)	(0.512)	(0.534)	(0.429)	(0.489)	(0.454)	(0.682)
Employees H	−0.151	−0.171	−0.207	−0.157	0.046	−0.048	−0.129
	(0.175)	(0.155)	(0.161)	(0.130)	(0.148)	(0.137)	(0.206)
Employees I	−0.272	−0.106	0.093	−0.116	0.085	0.145	−0.352
	(0.290)	(0.257)	(0.267)	(0.215)	(0.245)	(0.227)	(0.341)
Employees J	0.247	0.019	−0.052	0.0004	−0.111	−0.110	−0.281
	(0.390)	(0.345)	(0.360)	(0.289)	(0.330)	(0.306)	(0.460)
Employees L	0.841	0.385	0.336	0.030	−0.415	−0.638	0.030
	(0.596)	(0.528)	(0.550)	(0.442)	(0.504)	(0.468)	(0.703)
Employees M	−1.170**	−0.544	−0.156	−0.449	−0.233	0.079	−0.110
	(0.550)	(0.487)	(0.508)	(0.408)	(0.465)	(0.431)	(0.648)
Employees N	−0.075	0.120	0.036	0.212	0.352	0.263	0.340
	(0.500)	(0.442)	(0.461)	(0.370)	(0.423)	(0.392)	(0.589)
Employees P	−0.277	−0.187	−0.064	−0.021	−0.103	0.103	−0.232
	(0.184)	(0.163)	(0.169)	(0.136)	(0.155)	(0.144)	(0.216)
Employees Q	−0.060	−0.121	−0.022	−0.066	−0.054	−0.316	−0.017
	(0.281)	(0.249)	(0.259)	(0.208)	(0.238)	(0.220)	(0.331)
Employees R	0.139	0.122	0.153	0.207	0.003	−0.114	0.300
	(0.198)	(0.175)	(0.182)	(0.147)	(0.167)	(0.155)	(0.233)
Employees S	−0.008	0.188	0.096	0.216	0.017	−0.145	0.515
	(0.474)	(0.420)	(0.437)	(0.351)	(0.401)	(0.372)	(0.558)
nuts_ITH	−0.052	−0.199	−0.183	−0.038	0.092	0.244	−0.095
	(0.275)	(0.244)	(0.254)	(0.204)	(0.233)	(0.216)	(0.325)

nuts_ITI	−0.711***	−0.793***	−0.709***	−0.706***	−0.732***	−0.690***	−0.639***
	(0.189)	(0.168)	(0.175)	(0.140)	(0.160)	(0.149)	(0.223)
nuts_ITF	−0.737***	−0.845***	−0.484*	−0.376*	−0.497**	−0.634***	−0.436
	(0.271)	(0.240)	(0.250)	(0.201)	(0.229)	(0.212)	(0.319)
nuts_ITG	−0.385**	−0.381***	−0.394***	−0.869***	−1.282***	−1.938***	−1.152***
	(0.158)	(0.140)	(0.146)	(0.117)	(0.134)	(0.124)	(0.187)
Revenues per employee C:nuts_ITF	−0.063	−0.032	0.199	−0.113	−0.028	−0.106	0.068
	(0.255)	(0.226)	(0.235)	(0.189)	(0.216)	(0.200)	(0.300)
Revenues per employee F:nuts_ITF	0.315	0.108	0.152	−0.088	−0.186	−0.364*	−0.176
	(0.273)	(0.241)	(0.251)	(0.202)	(0.230)	(0.214)	(0.321)
Revenues per employee Q:nuts_ITF	−0.090	−0.019	−0.025	−0.183*	−0.053	−0.045	−0.040
	(0.133)	(0.118)	(0.123)	(0.098)	(0.112)	(0.104)	(0.157)
Revenues per employee R:nuts_ITF	−0.034	−0.247	−0.248	−0.540*	−0.620*	−0.318	−0.197
	(0.401)	(0.355)	(0.370)	(0.297)	(0.339)	(0.315)	(0.473)
Revenues per employee C:nuts_ITH	0.775*	0.888**	0.925**	0.290	0.374	0.157	0.710
	(0.406)	(0.359)	(0.374)	(0.301)	(0.343)	(0.318)	(0.478)
Revenues per employee F:nuts_ITH	0.239	0.098	0.025	−0.022	−0.259	−0.234	0.006
	(0.254)	(0.225)	(0.234)	(0.188)	(0.215)	(0.199)	(0.299)
Revenues per employee Q:nuts_ITH	−0.035	0.210	0.232	0.065	0.093	−0.038	0.149
	(0.167)	(0.148)	(0.154)	(0.124)	(0.141)	(0.131)	(0.197)
Revenues per employee R:nuts_ITH	−0.355	−0.184	−0.053	−0.577*	−0.644*	−0.230	−0.262
	(0.406)	(0.359)	(0.374)	(0.301)	(0.343)	(0.318)	(0.478)
Revenues per employee C:nuts_ITI	−0.334	−0.410*	−0.205	−0.124	0.078	0.445**	0.005
	(0.262)	(0.232)	(0.242)	(0.194)	(0.222)	(0.205)	(0.309)
Revenues per employee F:nuts_ITI	0.298	0.262	0.323	0.236	0.249	0.526***	0.171
	(0.218)	(0.193)	(0.202)	(0.162)	(0.185)	(0.171)	(0.257)
Revenues per employee Q:nuts_ITI	−0.166	−0.069	−0.153	−0.194*	−0.119	−0.084	−0.160
	(0.148)	(0.131)	(0.136)	(0.110)	(0.125)	(0.116)	(0.174)
Revenues per employee R:nuts_ITI	0.142	−0.103	0.015	−0.593*	−0.675*	−0.397	−0.189
	(0.428)	(0.379)	(0.395)	(0.318)	(0.362)	(0.336)	(0.505)
Revenues per employee C:nuts_ITG	−0.085	−0.044	0.049	−0.050	−0.073	−0.277***	−0.009
	(0.089)	(0.079)	(0.082)	(0.066)	(0.075)	(0.070)	(0.105)
Revenues per employee F:nuts_ITG	0.019	−0.095	−0.132	−0.765***	−1.331***	−2.135***	−1.020***
	(0.156)	(0.138)	(0.144)	(0.116)	(0.132)	(0.122)	(0.184)
Revenues per employee Q:nuts_ITG	0.002	0.047	0.074	0.051	0.068	0.173***	0.043
	(0.054)	(0.048)	(0.050)	(0.040)	(0.046)	(0.042)	(0.064)
Revenues per employee R:nuts_ITG	−0.218	−0.065	−0.174	−0.393**	−0.375**	−0.585***	−0.440*
	(0.212)	(0.188)	(0.196)	(0.157)	(0.179)	(0.166)	(0.250)
Constant	−0.308**	−0.072	−0.040	0.114	0.116	−0.021	−0.279
	(0.152)	(0.135)	(0.140)	(0.113)	(0.129)	(0.119)	(0.179)

AIC	1039.41	965.67	927.63	1067.09	1219.97	1348.98	1218.72
BIC	1287.47	1213.74	1175.7	1315.15	1468.04	1597.05	1098.72

Note: *p<0.1; **p<0.05; ***p<0.01

6.2 Results with standard errors obtained via wild bootstrap

Table 10. Quantile regression model with macroarea interactions

	Dependent variable: lockdown indicator						
	q10	q20	q30	q70	q80	q90	OLS
Income per capita	0.303***	0.234***	0.167**	0.243***	0.243**	0.169	0.208**
	(0.100)	(0.081)	(0.080)	(0.093)	(0.097)	(0.105)	(0.090)
Population density_2016	0.155***	0.136***	0.147***	0.226**	0.146**	0.109	0.179***
	(0.060)	(0.045)	(0.039)	(0.094)	(0.063)	(0.072)	(0.053)
Income Inequality	0.019	−0.054	−0.046	−0.071	−0.005	0.055	−0.059
	(0.045)	(0.042)	(0.042)	(0.049)	(0.057)	(0.056)	(0.047)
Revenue concentration	0.022	0.064*	0.095***	0.043	−0.007	0.033	0.051
	(0.042)	(0.036)	(0.035)	(0.044)	(0.047)	(0.067)	(0.053)
Essential workers	−0.011	−0.016	0.025	−0.003	−0.069	−0.018	−0.076
	(0.064)	(0.062)	(0.073)	(0.087)	(0.097)	(0.072)	(0.071)
Remote workers	0.134*	0.096	0.106	0.036	0.092	0.004	0.069
	(0.071)	(0.074)	(0.073)	(0.096)	(0.103)	(0.085)	(0.079)
Revenues per employee B	0.029	0.068	0.036	−0.030	−0.041	−0.037	−0.009
	(0.050)	(0.042)	(0.038)	(0.045)	(0.044)	(0.059)	(0.057)
Revenues per employee C	0.035	0.064	0.112	0.073	0.078	0.001	0.166
	(0.269)	(0.217)	(0.205)	(0.210)	(0.217)	(0.209)	(0.216)
Revenues per employee D	0.107**	0.071	0.026	−0.026	−0.006	−0.004	−0.020
	(0.048)	(0.045)	(0.038)	(0.044)	(0.038)	(0.044)	(0.054)
Revenues per employee E	0.031	0.026	−0.004	0.076	0.023	−0.014	0.019
	(0.073)	(0.064)	(0.064)	(0.059)	(0.068)	(0.080)	(0.068)
Revenues per employee F	0.009	0.028	−0.024	−0.033	−0.001	−0.388	−0.131
	(0.215)	(0.167)	(0.147)	(0.232)	(0.223)	(0.311)	(0.194)
Revenues per employee G	0.027	0.100	0.095	−0.028	0.004	−0.051	0.028
	(0.086)	(0.085)	(0.090)	(0.058)	(0.068)	(0.072)	(0.078)
Revenues per employee H	0.164**	0.131**	0.125**	0.007	0.067	−0.035	0.064
	(0.066)	(0.055)	(0.062)	(0.067)	(0.075)	(0.072)	(0.070)
Revenues per employee I	−0.040	−0.053	−0.095*	−0.136***	−0.140**	−0.116**	−0.082
	(0.050)	(0.053)	(0.051)	(0.048)	(0.061)	(0.057)	(0.057)
Revenues per employee J	−0.056	−0.023	−0.038	−0.028	0.017	−0.062	−0.047
	(0.053)	(0.050)	(0.058)	(0.051)	(0.065)	(0.071)	(0.055)
Revenues per employee L	0.115	0.226***	0.210***	0.162**	0.172*	0.235***	0.167*
	(0.101)	(0.073)	(0.069)	(0.077)	(0.090)	(0.078)	(0.088)
Revenues per employee M	−0.084	−0.132	−0.126	−0.118	−0.141	−0.044	−0.136
	(0.122)	(0.093)	(0.092)	(0.121)	(0.118)	(0.095)	(0.113)
Revenues per employee N	0.129***	0.090**	0.064	0.003	−0.012	−0.067	0.044
	(0.041)	(0.042)	(0.044)	(0.039)	(0.048)	(0.060)	(0.050)

Revenues per employee P	−0.174***	−0.085	−0.086	−0.064	−0.101	−0.025	−0.084
	(0.064)	(0.064)	(0.066)	(0.086)	(0.090)	(0.088)	(0.072)
Revenues per employee Q	−0.139	−0.150**	−0.071	−0.011	−0.077	0.006	−0.075
	(0.125)	(0.074)	(0.069)	(0.097)	(0.115)	(0.130)	(0.112)
Revenues per employee R	−0.016	−0.041	−0.181	−0.117	0.298	0.358	−0.125
	(0.466)	(0.371)	(0.318)	(0.400)	(0.537)	(0.463)	(0.420)
Revenues per employee S	0.003	−0.023	−0.002	−0.096	−0.173*	−0.259**	−0.127
	(0.079)	(0.062)	(0.068)	(0.076)	(0.101)	(0.108)	(0.081)
Employees B	−0.207	−0.230	−0.145	−0.006	−0.056	−0.092	−0.182
	(0.188)	(0.158)	(0.128)	(0.112)	(0.121)	(0.129)	(0.142)
Employees C	0.372*	0.414**	0.323**	−0.140	−0.022	0.129	0.221
	(0.202)	(0.161)	(0.149)	(0.144)	(0.158)	(0.161)	(0.174)
Employees D	−0.343	−0.592**	−0.646***	−0.616**	−0.265	−0.128	−0.406
	(0.345)	(0.241)	(0.230)	(0.254)	(0.253)	(0.210)	(0.257)
Employees E	−0.016	0.205	0.150	0.105	−0.242	−0.218	−0.199
	(0.240)	(0.172)	(0.204)	(0.233)	(0.253)	(0.182)	(0.227)
Employees F	−0.021	−0.170	−0.067	0.041	0.323	0.068	−0.024
	(0.453)	(0.283)	(0.312)	(0.365)	(0.448)	(0.421)	(0.379)
Employees G	1.762*	0.628	0.329	1.140	0.696	0.045	0.345
	(1.003)	(0.727)	(0.632)	(0.832)	(0.870)	(0.694)	(0.708)
Employees H	−0.328	−0.379**	−0.374**	−0.415**	0.045	0.199	−0.238
	(0.269)	(0.149)	(0.149)	(0.189)	(0.359)	(0.317)	(0.214)
Employees I	−1.347**	−0.751*	−0.186	0.044	−0.059	−0.093	−0.442
	(0.567)	(0.425)	(0.350)	(0.288)	(0.354)	(0.354)	(0.355)
Employees J	0.724	0.440	0.180	0.308	0.003	−0.396	−0.046
	(0.776)	(0.514)	(0.437)	(0.427)	(0.478)	(0.413)	(0.478)
Employees L	1.897**	1.513**	1.233*	0.110	0.011	−0.402	0.505
	(0.894)	(0.701)	(0.686)	(0.669)	(0.655)	(0.731)	(0.730)
Employees M	−2.185**	−1.090	−0.696	−0.585	−0.385	0.552	−0.382
	(1.007)	(0.780)	(0.638)	(0.630)	(0.654)	(0.608)	(0.674)
Employees N	−0.290	−0.491	−0.523	−0.698	−0.649	−0.131	−0.265
	(0.772)	(0.566)	(0.472)	(0.554)	(0.598)	(0.506)	(0.612)
Employees P	−0.697*	−0.162	−0.075	−0.051	−0.088	−0.147	−0.085
	(0.392)	(0.278)	(0.222)	(0.294)	(0.242)	(0.194)	(0.225)
Employees Q	−0.373	−0.171	−0.070	0.099	0.056	−0.099	−0.056
	(0.391)	(0.277)	(0.266)	(0.416)	(0.390)	(0.354)	(0.344)
Employees R	0.882***	0.731***	0.411*	0.143	0.165	0.327	0.500**
	(0.262)	(0.222)	(0.223)	(0.229)	(0.251)	(0.277)	(0.242)
Employees S	0.180	0.166	0.236	0.544	0.459	0.344	0.828
	(0.638)	(0.511)	(0.474)	(0.578)	(0.565)	(0.479)	(0.580)

nuts_ITH	0.872***	0.421	0.771***	0.723***	0.486*	0.349	0.550
	(0.268)	(0.266)	(0.276)	(0.260)	(0.276)	(0.261)	(0.337)
nuts_ITI	−0.047	−0.095	−0.139	−0.264	−0.351	−0.030	−0.227
	(0.218)	(0.173)	(0.178)	(0.209)	(0.248)	(0.224)	(0.232)
nuts_ITF	0.455	0.215	−0.099	0.220	0.386	0.921**	0.154
	(0.334)	(0.297)	(0.341)	(0.387)	(0.412)	(0.444)	(0.332)
nuts_ITG	0.237	0.147	0.071	−0.191	−0.247	−1.025*	−0.253
	(0.176)	(0.176)	(0.209)	(0.317)	(0.311)	(0.547)	(0.194)
Revenues per employee C:nuts_ITF	−0.0002	−0.225	−0.175	−0.062	−0.225	0.158	−0.197
	(0.346)	(0.350)	(0.377)	(0.332)	(0.308)	(0.324)	(0.312)
Revenues per employee F:nuts_ITF	0.082	−0.135	−0.221	−0.005	0.113	0.485	−0.155
	(0.317)	(0.347)	(0.414)	(0.385)	(0.367)	(0.368)	(0.334)
Revenues per employee Q:nuts_ITF	0.208	0.324***	0.167	−0.060	0.183	0.263	0.159
	(0.165)	(0.112)	(0.117)	(0.172)	(0.208)	(0.200)	(0.163)
Revenues per employee R:nuts_ITF	0.095	0.122	0.209	0.367	−0.043	0.154	0.296
	(0.558)	(0.425)	(0.369)	(0.446)	(0.588)	(0.528)	(0.491)
Revenues per employee C:nuts_ITH	0.363	0.388	1.247***	0.571	0.278	0.209	0.582
	(0.514)	(0.587)	(0.459)	(0.402)	(0.434)	(0.392)	(0.497)
Revenues per employee F:nuts_ITH	−0.965***	−0.803***	−0.760***	−0.413	−0.334	−0.120	−0.444
	(0.308)	(0.260)	(0.253)	(0.276)	(0.280)	(0.238)	(0.311)
Revenues per employee Q:nuts_ITH	0.986***	0.938***	0.508**	0.058	0.176	0.083	0.387*
	(0.221)	(0.209)	(0.220)	(0.162)	(0.196)	(0.198)	(0.205)
Revenues per employee R:nuts_ITH	−0.031	−0.167	0.153	0.117	−0.399	−0.461	0.004
	(0.558)	(0.412)	(0.368)	(0.451)	(0.592)	(0.527)	(0.497)
Revenues per employee C:nuts_ITI	−0.235	−0.198	−0.128	0.149	0.293	0.840**	0.020
	(0.355)	(0.280)	(0.265)	(0.287)	(0.339)	(0.340)	(0.321)
Revenues per employee F:nuts_ITI	−0.295	−0.465**	−0.312	0.135	0.117	0.449	−0.129
	(0.280)	(0.202)	(0.199)	(0.227)	(0.239)	(0.287)	(0.267)
Revenues per employee Q:nuts_ITI	0.106	0.040	−0.023	0.025	0.042	−0.135	0.048
	(0.188)	(0.126)	(0.120)	(0.150)	(0.163)	(0.221)	(0.181)
Revenues per employee R:nuts_ITI	0.103	0.041	0.206	0.123	−0.437	−0.108	0.143
	(0.606)	(0.434)	(0.377)	(0.482)	(0.608)	(0.629)	(0.525)
Revenues per employee C:nuts_ITG	0.079	0.075	0.048	−0.004	−0.011	−0.152	−0.040
	(0.126)	(0.108)	(0.103)	(0.116)	(0.119)	(0.115)	(0.109)
Revenues per employee F:nuts_ITG	−0.058	−0.099	−0.017	−0.468	−0.571*	−1.652***	−0.568***
	(0.174)	(0.180)	(0.192)	(0.328)	(0.328)	(0.610)	(0.191)
Revenues per employee Q:nuts_ITG	0.155*	0.160***	0.104	0.089	0.145*	0.164**	0.185***
	(0.079)	(0.058)	(0.063)	(0.062)	(0.076)	(0.082)	(0.066)
Revenues per employee R:nuts_ITG	0.152	−0.009	0.031	−0.017	−0.252	−0.097	−0.027
	(0.332)	(0.366)	(0.366)	(0.373)	(0.375)	(0.271)	(0.259)

Constant	-0.868^{***}	-0.628^{***}	-0.428^{***}	0.039	0.223	0.073	-0.269
	(0.181)	(0.145)	(0.152)	(0.171)	(0.201)	(0.290)	(0.186)
AIC	1077.58	1072.87	1108.63	1261.38	1373.51	1453.74	1256.68
BIC	1325.65	1320.94	1356.7	1509.45	1621.58	1701.81	1136.68

Note: *p<0.1; **p<0.05; ***p<0.01

Table 11. Quantile regression model with macroarea interactions

	Dependent variable: recovery indicator						
	q10	q20	q30	q70	q80	q90	OLS
Income per capita	0.226***	0.182***	0.162**	0.217***	0.223***	0.281***	0.217**
	(0.077)	(0.069)	(0.063)	(0.061)	(0.062)	(0.096)	(0.087)
Population density_2016	0.119***	0.101***	0.082***	0.103**	0.118*	0.080	0.125**
	(0.028)	(0.037)	(0.027)	(0.045)	(0.061)	(0.063)	(0.051)
Income Inequality	0.004	−0.040	−0.086***	−0.107***	−0.110***	−0.090*	−0.079*
	(0.041)	(0.032)	(0.032)	(0.031)	(0.033)	(0.048)	(0.046)
Revenue concentration	0.075**	0.056*	0.065**	0.029	0.031	−0.031	0.041
	(0.036)	(0.033)	(0.029)	(0.030)	(0.038)	(0.045)	(0.051)
Essential workers	−0.077	−0.107*	−0.026	−0.129**	−0.125*	−0.178**	−0.193***
	(0.060)	(0.057)	(0.061)	(0.065)	(0.066)	(0.069)	(0.068)
Remote workers	0.148*	0.145**	0.090	0.078	0.051	−0.012	0.082
	(0.076)	(0.064)	(0.064)	(0.067)	(0.069)	(0.081)	(0.076)
Revenues per employee B	0.035	0.025	−0.018	0.018	0.020	0.086	0.066
	(0.060)	(0.053)	(0.034)	(0.037)	(0.035)	(0.057)	(0.054)
Revenues per employee C	0.328*	0.161	−0.007	0.092	−0.075	0.074	0.024
	(0.184)	(0.152)	(0.153)	(0.119)	(0.140)	(0.176)	(0.208)
Revenues per employee D	0.028	0.011	−0.010	−0.037	−0.005	−0.062	−0.038
	(0.060)	(0.047)	(0.038)	(0.029)	(0.033)	(0.048)	(0.052)
Revenues per employee E	0.084	0.096	0.152***	−0.013	−0.016	−0.025	0.025
	(0.071)	(0.067)	(0.054)	(0.043)	(0.051)	(0.061)	(0.065)
Revenues per employee F	−0.475***	−0.556***	−0.566***	−0.386**	−0.400*	−0.603*	−0.566***
	(0.164)	(0.150)	(0.162)	(0.178)	(0.236)	(0.326)	(0.186)
Revenues per employee G	−0.168**	−0.046	−0.007	−0.053	−0.100*	−0.208**	−0.065
	(0.075)	(0.073)	(0.067)	(0.044)	(0.054)	(0.086)	(0.075)
Revenues per employee H	0.048	0.134**	0.140***	0.065	0.061	−0.059	0.053
	(0.061)	(0.060)	(0.053)	(0.045)	(0.052)	(0.065)	(0.067)
Revenues per employee I	0.102*	0.085**	0.054	−0.057*	−0.095**	−0.095*	−0.007
	(0.053)	(0.042)	(0.037)	(0.033)	(0.037)	(0.050)	(0.055)
Revenues per employee J	−0.088*	−0.040	−0.050	−0.013	0.026	−0.015	−0.066
	(0.049)	(0.041)	(0.039)	(0.037)	(0.044)	(0.054)	(0.053)
Revenues per employee L	0.257***	0.254***	0.237***	0.182***	0.178***	0.206***	0.206**
	(0.081)	(0.079)	(0.060)	(0.053)	(0.065)	(0.073)	(0.085)
Revenues per employee M	−0.032	−0.023	−0.088	−0.107	−0.036	0.047	−0.165
	(0.103)	(0.093)	(0.062)	(0.069)	(0.076)	(0.101)	(0.108)
Revenues per employee N	0.026	0.049	0.102**	0.053	0.012	0.026	0.055
	(0.043)	(0.044)	(0.046)	(0.037)	(0.036)	(0.063)	(0.048)
Revenues per employee P	−0.022	−0.012	0.050	−0.024	−0.077	−0.058	−0.041
	(0.060)	(0.059)	(0.053)	(0.047)	(0.057)	(0.070)	(0.069)

Revenues per employee Q	0.179	0.040	0.049	0.030	−0.019	0.035	0.061
	(0.123)	(0.089)	(0.059)	(0.059)	(0.075)	(0.102)	(0.107)
Revenues per employee R	−0.158	0.163	0.042	0.396	0.523	−0.013	−0.010
	(0.345)	(0.310)	(0.294)	(0.335)	(0.346)	(0.439)	(0.404)
Revenues per employee S	−0.098	−0.077	−0.015	−0.037	−0.160**	−0.260**	−0.018
	(0.074)	(0.068)	(0.068)	(0.060)	(0.076)	(0.102)	(0.078)
Employees B	−0.042	−0.075	−0.079	−0.083	−0.065	−0.071	−0.164
	(0.121)	(0.111)	(0.079)	(0.070)	(0.072)	(0.138)	(0.137)
Employees C	0.160	0.092	0.104	−0.055	−0.179*	−0.195	−0.019
	(0.126)	(0.145)	(0.108)	(0.090)	(0.098)	(0.139)	(0.168)
Employees D	−0.303	−0.242	−0.415**	−0.321**	−0.189	−0.036	−0.222
	(0.229)	(0.229)	(0.173)	(0.126)	(0.145)	(0.178)	(0.248)
Employees E	0.024	0.002	−0.038	−0.065	−0.165	−0.083	−0.066
	(0.161)	(0.190)	(0.174)	(0.135)	(0.156)	(0.163)	(0.218)
Employees F	0.041	0.155	0.211	0.087	0.294	0.525	0.209
	(0.265)	(0.277)	(0.268)	(0.198)	(0.263)	(0.429)	(0.364)
Employees G	0.944	0.378	0.046	0.541	0.627	0.571	0.192
	(0.588)	(0.548)	(0.405)	(0.405)	(0.490)	(0.640)	(0.682)
Employees H	−0.151	−0.171	−0.207**	−0.157	0.046	−0.048	−0.129
	(0.163)	(0.162)	(0.103)	(0.098)	(0.182)	(0.231)	(0.206)
Employees I	−0.272	−0.106	0.093	−0.116	0.085	0.145	−0.352
	(0.377)	(0.351)	(0.254)	(0.188)	(0.217)	(0.381)	(0.341)
Employees J	0.247	0.019	−0.052	0.0004	−0.111	−0.110	−0.281
	(0.434)	(0.395)	(0.255)	(0.228)	(0.247)	(0.372)	(0.460)
Employees L	0.841*	0.385	0.336	0.030	−0.415	−0.638	0.030
	(0.482)	(0.524)	(0.435)	(0.355)	(0.430)	(0.575)	(0.703)
Employees M	−1.170*	−0.544	−0.156	−0.449	−0.233	0.079	−0.110
	(0.648)	(0.578)	(0.392)	(0.320)	(0.406)	(0.530)	(0.648)
Employees N	−0.075	0.120	0.036	0.212	0.352	0.263	0.340
	(0.400)	(0.403)	(0.374)	(0.383)	(0.303)	(0.401)	(0.589)
Employees P	−0.277	−0.187	−0.064	−0.021	−0.103	0.103	−0.232
	(0.238)	(0.225)	(0.128)	(0.171)	(0.182)	(0.250)	(0.216)
Employees Q	−0.060	−0.121	−0.022	−0.066	−0.054	−0.316	−0.017
	(0.312)	(0.287)	(0.207)	(0.251)	(0.283)	(0.322)	(0.331)
Employees R	0.139	0.122	0.153	0.207	0.003	−0.114	0.300
	(0.211)	(0.194)	(0.169)	(0.149)	(0.142)	(0.171)	(0.233)
Employees S	−0.008	0.188	0.096	0.216	0.017	−0.145	0.515
	(0.438)	(0.480)	(0.381)	(0.337)	(0.359)	(0.397)	(0.558)
nuts_ITH	−0.052	−0.199	−0.183	−0.038	0.092	0.244	−0.095
	(0.345)	(0.254)	(0.225)	(0.161)	(0.175)	(0.244)	(0.325)

nuts_ITI	-0.711^{***}	-0.793^{***}	-0.709^{***}	-0.706^{***}	-0.732^{***}	-0.690^{***}	-0.639^{***}
	(0.218)	(0.160)	(0.126)	(0.134)	(0.153)	(0.213)	(0.223)
nuts_ITF	-0.737^{**}	-0.845^{***}	-0.484^{*}	-0.376^{*}	-0.497^{**}	-0.634^{*}	-0.436
	(0.316)	(0.306)	(0.286)	(0.205)	(0.250)	(0.370)	(0.319)
nuts_ITG	-0.385^{**}	-0.381^{**}	-0.394^{**}	-0.869^{***}	-1.282^{***}	-1.938^{***}	-1.152^{***}
	(0.179)	(0.168)	(0.169)	(0.227)	(0.411)	(0.441)	(0.187)
Revenues per employee C:nuts_ITF	-0.063	-0.032	0.199	-0.113	-0.028	-0.106	0.068
	(0.286)	(0.351)	(0.315)	(0.228)	(0.202)	(0.255)	(0.300)
Revenues per employee F:nuts_ITF	0.315	0.108	0.152	-0.088	-0.186	-0.364	-0.176
	(0.305)	(0.350)	(0.352)	(0.275)	(0.261)	(0.373)	(0.321)
Revenues per employee Q:nuts_ITF	-0.090	-0.019	-0.025	-0.183	-0.053	-0.045	-0.040
	(0.174)	(0.141)	(0.094)	(0.142)	(0.146)	(0.150)	(0.157)
Revenues per employee R:nuts_ITF	-0.034	-0.247	-0.248	-0.540	-0.620^{*}	-0.318	-0.197
	(0.401)	(0.361)	(0.354)	(0.351)	(0.371)	(0.471)	(0.473)
Revenues per employee C:nuts_ITH	0.775	0.888^{***}	0.925^{***}	0.290	0.374	0.157	0.710
	(0.488)	(0.340)	(0.324)	(0.253)	(0.254)	(0.312)	(0.478)
Revenues per employee F:nuts_ITH	0.239	0.098	0.025	-0.022	-0.259	-0.234	0.006
	(0.301)	(0.257)	(0.250)	(0.196)	(0.180)	(0.235)	(0.299)
Revenues per employee Q:nuts_ITH	-0.035	0.210	0.232	0.065	0.093	-0.038	0.149
	(0.241)	(0.209)	(0.187)	(0.097)	(0.117)	(0.152)	(0.197)
Revenues per employee R:nuts_ITH	-0.355	-0.184	-0.053	-0.577	-0.644^{*}	-0.230	-0.262
	(0.424)	(0.348)	(0.338)	(0.367)	(0.380)	(0.481)	(0.478)
Revenues per employee C:nuts_ITI	-0.334	-0.410^{*}	-0.205	-0.124	0.078	0.445	0.005
	(0.256)	(0.215)	(0.216)	(0.188)	(0.198)	(0.299)	(0.309)
Revenues per employee F:nuts_ITI	0.298	0.262	0.323^{*}	0.236	0.249	0.526^{**}	0.171
	(0.181)	(0.180)	(0.181)	(0.179)	(0.176)	(0.264)	(0.257)
Revenues per employee Q:nuts_ITI	-0.166	-0.069	-0.153^{*}	-0.194^{**}	-0.119	-0.084	-0.160
	(0.172)	(0.141)	(0.089)	(0.094)	(0.118)	(0.167)	(0.174)
Revenues per employee R:nuts_ITI	0.142	-0.103	0.015	-0.593	-0.675^{*}	-0.397	-0.189
	(0.427)	(0.376)	(0.364)	(0.384)	(0.397)	(0.516)	(0.505)
Revenues per employee C:nuts_ITG	-0.085	-0.044	0.049	-0.050	-0.073	-0.277^{***}	-0.009
	(0.089)	(0.075)	(0.072)	(0.070)	(0.079)	(0.107)	(0.105)
Revenues per employee F:nuts_ITG	0.019	-0.095	-0.132	-0.765^{***}	-1.331^{***}	-2.135^{***}	-1.020^{***}
	(0.186)	(0.164)	(0.175)	(0.264)	(0.463)	(0.641)	(0.184)
Revenues per employee Q:nuts_ITG	0.002	0.047	0.074^{*}	0.051	0.068	0.173^{*}	0.043
	(0.072)	(0.053)	(0.042)	(0.052)	(0.072)	(0.092)	(0.064)
Revenues per employee R:nuts_ITG	-0.218	-0.065	-0.174	-0.393	-0.375	-0.585	-0.440^{*}
	(0.295)	(0.255)	(0.205)	(0.298)	(0.285)	(0.371)	(0.250)
Constant	-0.308^{*}	-0.072	-0.040	0.114	0.116	-0.021	-0.279
	(0.173)	(0.136)	(0.133)	(0.126)	(0.194)	(0.273)	(0.179)

AIC	1039.41	965.67	927.63	1067.09	1219.97	1348.98	1218.72
BIC	1287.47	1213.74	1175.7	1315.15	1468.04	1597.05	1098.72
Note:							*p<0.1; **p<0.05; ***p<0.01

References

1. Barabási, A.-L. *et al.* *Network science* (Cambridge university press, 2016).

2. Newman, M. *Networks* (Oxford university press, 2018).

3. Feng, X., He, X. & Hu, J. Wild bootstrap for quantile regression. *Biometrika* **98**, 995–999 (2011).
